# Phenotyping fatty acids and the *trans*-10 shift in the rumen content and adipose tissue of commercial finishing lambs

**DOI:** 10.1016/j.vas.2026.100662

**Published:** 2026-04-15

**Authors:** Chica Manuel, Cristina Xavier, Saeedeh Moradi, Diana M. Soares, Aristides Manuel, Liliana Cachucho, Letícia Fialho, David Soldado, Andreia Silva, Patrícia Lage, Olinda Guerreiro, Cármen Garrine Bule, Eliana Jerónimo, Rui J.B. Bessa, Susana P. Alves

**Affiliations:** a UNIZAMBEZE – Zambeze University, Faculty of Agricultural and Forestry Engineering, Mozambique; b CIISA – Centre for Interdisciplinary Research in Animal Health, Faculty of Veterinary Medicine, University of Lisbon, Lisbon, Portugal; c Associate Laboratory for Animal and Veterinary Sciences (AL4AnimalS), Portugal; d Centro de Biotecnologia Agrícola e Agro-Alimentar do Alentejo (CEBAL), Instituto Politécnico de Beja (IPBeja), 7801-908 Beja, Portugal; e MED – Mediterranean Institute for Agriculture, Environment and Development & CHANGE – Institute for Global Changes and Sustainability, CEBAL, 7801-908 Beja, Portugal; f Faculdade de Veterinária, Departamento de Produção Animal, Universidade Eduardo Mondlane, 3453 Avenida Julius Nyerere, Maputo, Mozambique

**Keywords:** *Trans*-10 shift, Lambs, Rumen, Fatty acids, Dimethyl acetals, Genetic group

## Abstract

The *trans*-10 shifted ruminal biohydrogenation (BH) of unsaturated fatty acids (FA) pathways is characterised by the replacement of *trans*-11 18:1 by *trans*-10 18:1 as the predominant ruminal BH intermediate. The *t*10-shift occurs frequently in animals kept in intensive feeding systems and affects the nutritional quality of ruminant-derived foods. This study aimed to phenotype the prevalence and variability of the *t*10-shift in a large population of finishing lambs and to relate it to rumen fermentation parameters, microbial lipid markers, and adipose tissue FA. A total of 630 lambs from seven genetic groups were finished in a lamb finishing unit on a high-concentrate diet. The sampling covered 11 collection occasions at the slaughterhouse between 2022 and 2024. Rumen digesta, collected post-mortem, were analysed for volatile fatty acids (VFA), long-chain FA, and dimethyl acetals (DMA), while kidney knob channel fat (KKCF) was analysed in a subset of animals (*n* = 111). The *t*10-shift ratio (*t*10-18:1/*t*11-18:1) in the rumen content was >1 for 89% of the animals. Significant genetic group effects were observed for rumen pH, VFA, DMA, and FA composition, including C18 intermediates. *Trans*-10 18:1 and *t*10-shift ratio were negatively correlated with rumen pH and 18:0, indicating more incomplete ruminal BH pathways under acidic conditions. In KKCF, differences in *trans*-18:1 isomers were detected among groups, and rumen *t*10-18:1 and *t*11-18:1 were positively associated with the deposition of *t*10-18:1 and *t*11-18:1 + *c*9*t*11-18:2 in adipose tissue, respectively. Notably, a minority of animals appeared resistant to the *t*10-shift despite standardised finishing conditions, highlighting potential for genetic selection.

## 1 Introduction

Dietary lipids are metabolised by rumen microbes through lipolysis and biohydrogenation (BH) in the rumen. During lipolysis, lipids are hydrolysed by microbial enzymes, releasing free fatty acids (FA), which are then available for BH, a process involving isomerisation and hydrogenation of FA double bonds, transforming dietary unsaturated FA into *trans* and saturated FA in the rumen (Harfoot & Hazlewood, 1997; Lourenço et al., 2010). In intensive ruminant production systems, diets rich in highly fermentable carbohydrates can lower rumen pH and alter the composition and activity of the rumen microbiota, thereby affecting ruminal BH pathways (Dewanckele et al., 2020b; Enjalbert et al., 2023). In most dietary conditions, rumen BH pathways produce vaccenic acid (*trans*-11 18:1 or *t*11-18:1) as the main intermediate, but when the incorporation of concentrates increases in the diets, the main BH intermediate formed in the rumen tends to be the *trans*-10 18:1 that often replaces the *trans*-11 18:1 as the main BH intermediate. Thus, we consider that the “*trans*-10 shift” occurs in the rumen when the ratio of *trans*-10 18:1 to *trans*-11 18:1 exceeds 1 (Alves et al., 2021; Bessa et al., 2015). A high flow of *trans*-11 18:1 to the small intestine is desired, as after absorption, it can be converted into rumenic acid (*cis*-9*trans*-11-18:2 or *c*9*t*11-18:2) by the action of the delta-9 desaturase enzyme in tissues. The *c*9*t*11-18:2 is the main conjugated linoleic acid (CLA) isomer present in ruminants, and several studies have reported that it is a potential bioactive lipid that enhances the nutritional quality of milk and meat, providing benefits to human health (Badawy et al., 2023; Vahmani et al., 2020; Xu et al., 2024). Thus, dietary strategies to increase the rumen outflow of *trans*-11 18:1 and, hence, the concentration of *c*9*t*11-CLA in ruminant edible products have been extensively researched. However, those dietary strategies fail when the *trans*-10 shifted BH pathway is established in the rumen, because *trans*-10 18:1, after absorption in the small intestine, is not converted into CLA and accumulates in tissues (Bessa et al., 2015). Additionally, studies suggest that high intakes of *trans*-18:1 from ruminant fats may have biological effects similar to those of *trans*-18:1 from partially hydrogenated vegetable oil (industrial *trans*), including contributing to the development of cardiovascular disease (CVD) (Alves et al., 2021; Gebauer et al., 2015; Vahmani et al., 2025). Therefore, research on formulating diets that prevent the occurrence of the *trans*-10 shift, and the subsequent accumulation of *trans*-10 18:1 in ruminant edible fats, while maintaining productivity comparable to that of current high-concentrate diets, is necessary and has been extensively researched (Doreau et al., 2016; Santos-Silva et al., 2020, 2023).

It is clear that animals display a large individual variability regarding their susceptibility to express the *trans*-10 shift when fed high-concentrate diets (Bessa et al., 2015). This variability suggests that selecting animals that are less susceptible to the *trans*-10 shift could be a strategy to improve both animal productivity and the nutritional quality of ruminant-derived foods.

Several factors, such as genetic influences, variations in feeding behaviour, and feeding background, among others, may contribute to individual differences in the microbiome (Khiaosa-ard et al., 2014) and to the expression of the *trans*-10 shift (Costa et al., 2013b). To better understand the main drivers of individual susceptibility to the *trans*-10 shift, more comprehensive studies are needed. Despite that, most of the data on individual variation to *trans*-10 shift trait derive from experiments with a small number of animals, from distinct finishing systems, or do not use the rumen content as the sample matrix but the meat or subcutaneous fat (Bessa et al., 2015; Bravo-Lamas et al., 2016; Santos-Silva et al., 2020, 2023). Therefore, the goal of this work was to phenotype the occurrence of the *trans*-10 shift in the rumen over two years, involving a large number of animals from different origins and genetic backgrounds, and finished under similar conditions. The research was also expanded to provide a comprehensive analysis of the FA composition, including microbial-derived FA and dimethyl acetals (DMA), in both rumen digesta and adipose tissue.

## 2 Materials and methods

### 2.1 Animals and sample collection

The study included samples from 630 female lambs from 23 producers in the Baixo Alentejo region of Portugal, finished in the same farm but originating from seven different counties (Serpa, Beja, Almodovar, Mértola, Castro Verde, Ourique, and Odemira), and distributed from seven genetic groups comprising purebred or crossed (X) lambs (Merino, Romane, X-ILFrance, Merino×Lacaune, X-Romane, X-Merino, and X-Suffolk). The crossbred animals resulted from crossing local ewes without breed (primarily derived from original autochthonous Merino Branco populations) with purebred males. The samples were collected from June 2022 to July 2024, resulting in 11 collection periods. Description of the study population, sampling design, and sampling dates are presented in Supplementary Table S1.

Feed management prior to arrival at the finishing farm included grazing on pasture. When pasture or forage was unavailable, the animals received supplemental concentrate feed and hay. According to records, most producers provide concentrated feed to lambs before and after weaning, typically at 45–60 days of age. Records about feed management are presented as Supplementary Table S2.

After arrival at the finishing farm (Carlos & Helder Alves, Sociedade Agro-Pecuária, LDA), lambs were housed indoors in pens that could accommodate approximately 100 animals and were ear tagged to facilitate their tracking into the commercial slaughterhouse. They were separated by sex and weight and subjected to consistent sanitation and feeding practices. Lambs were gradually transitioned to a commercial concentrate diet for lamb fattening, followed by continued feeding ad libitum of the commercial concentrate and straw in a 90:10 ratio for about 45 to 50 days before slaughter. The composition of the concentrate included 89.5 % of dry matter (DM), and 16.9 % DM of crude protein, 2.3 % DM of ether extract, 20.7 % DM of neutral detergent fiber (NDF), 8.9 % DM of acid detergent fiber (ADF), 2.4 % DM of acid detergent lignin (ADL), 46.0 % DM of starch, 6.3 % DM of sugars, and 7.3 % DM of ash.

After the finishing period, animals were transported to the commercial slaughterhouse in Odemira (Portugal), specifically at Matadouro of Litoral Alentejano, the day before slaughter (around 18–20 PM) and were slaughtered the morning before (around 7–9 PM). At the abattoir, animals have access only to water, so they were fasted for approximately 12 to 15 h before slaughter. Animals were sacrificed by exsanguination after being stunned in compliance with European regulations (EU, 2009). The whole rumen content of each animal was immediately collected and homogenized. A portion was rapidly frozen in dry ice, while another was filtered through four layers of cheesecloth to measure pH. An aliquot of 5 mL of rumen fluid was also transferred into a tube containing 1 mL of 25 % metaphosphoric acid and immediately frozen in dry ice for analysis of volatile fatty acids (VFA). Samples from the kidney knob channel fat (KKCF) were collected at the abattoir, but only a subset of samples (*n* = 111; Merino=43, Romane=14; X-ILFrance= 25; X-Merino=6, X-Romane=11, X-Suffolk=12) were used for FA analysis. The selection criterion for these samples was based on their fatty acid profile in rumen digesta, ensuring that animals with both high and low *t*10-shift were included. The cold carcass weight was also measured at the abattoir.

### 2.2 pH and volatile fatty acids

The pH of rumen fluid was immediately measured at the abattoir using a pH meter (pH-2005 Selecta). The VFA were measured by gas chromatography (GC) using iso-6:0 as the internal standard (50 mmol/L). The GC (Shimadzu 2010 Plus, Kyoto, Japan) was equipped with a flame ionization detector and a fused capillary column (Nukol, 30 m, 0.25 mm internal diameter, and 0.25 µm film thickness, Supelco, Bellefonte, PA, USA). The carrier gas was helium, 1 µL of sample was injected, and the split ratio was 1:50. The GC conditions included the injector temperature and the detector temperatures set to 250 °C and 280 °C, respectively. The column oven temperature was programmed to be isothermally at 180 °C for 10 min. Calibration curves for each VFA, using pure commercial standards, were constructed at concentrations ranging from 0.2 to 30 mmol/L, with iso-6:0 as the internal standard at 5 mmol/L, as described by Oliveira et al. (2016).

### 2.3 Fatty acid analysis

Before analysis, rumen content samples were lyophilised using a Scanvac Coolsafe freeze dryer (Scanvac, Denmark). Then, FA methyl esters were prepared from 0.250 g of freeze-dried samples according to Alves et al. (2013). Briefly, 1 mL of dry toluene and 1 mL of internal standard (19:0, at a concentration of 1 mg/mL) were added to the tubes containing the rumen digesta. Next, 2 mL of 0.5 M sodium methoxide solution in methanol was added, and the mixture was incubated in a water bath at 50 °C for 15 min. Afterwards, 3 mL of 1.25 M HCl in methanol was added, and the mixture was heated in a water bath at 80 °C for 20 min. After cooling to room temperature, 2 mL of 6 % aqueous K_2_CO_3_ solution and 4 mL of n-hexane were added. The supernatant was collected into a new tube along with 0.5 g of anhydrous sodium sulphate to remove water. The hexane phase containing the FA methyl esters evaporated in a stream of nitrogen at 37 °C. The extracts containing the FA methyl esters were redissolved in 1 mL of n-Hexane and transferred to GC vials and stored at -20 °C until analysis by GC. Samples from KKCF were also transesterified to prepare FA methyl esters with a similar procedure as described above, but with adjustments on the quantity of sample and volume of the basic and acid catalyst, as described in Alves et al. (2015).

### 2.4 Gas chromatography analyses

The FA methyl esters were separated and quantified using a Shimadzu 2010-Plus GC (Shimadzu, Kyoto, Japan) with a flame ionisation detector (FID) and a SP-2560 fused silica capillary column (100 m x 0.25 mm x 0.20 μm, Supelco, Bellefonte, PA, USA). The GC conditions were as follows: the injector and detector temperatures were maintained at 220 °C and 250 °C, respectively. Helium was used as the carrier gas at a constant flow rate of 1.0 mL/min, and 1 μL of the sample was injected at a split ratio of 1:50. The oven temperature was programmed to begin at 50 °C for 1 min, then increase to 150 °C at a rate of 50 °C/min and held at this temperature for 20 min. Subsequently, the temperature was raised to 190 °C at 1 °C/min, then to 220 °C at 2 °C/min, and held at 220 °C for a total of 118 min. Fatty acid identification was performed by comparing retention times with mixtures of commercially available standards (Supelco 37 Component FAME Mix, Supelco, Bellefonte, PA, USA), with an in-house library, and with published chromatograms (Alves et al., 2013).

### 2.5 Statistical analysis

The statistical analysis was conducted in SAS 9.4 (SAS Institute Inc., Cary, NC, USA) using PROC MIXED, with the genetic group as a fixed factor and cold carcass weight as a covariate. For each variable, the homogeneity of variances was also evaluated, and when needed, the heterogeneity of variances was accommodated in the model using the group option (group=genetic group) of the repeated statement of Proc Mixed. Differences between groups were considered when the *P*-value < 0.05, and when detected, multiple comparisons of means were conducted using the Tukey adjustment.

Since sample sizes were unequal in each group (rumen content samples: *n* = 611; Merino=250; Romane=43; X-ILFrance=134; X-Merino=49; X-Romane=80; X-Suffolk=41; Merino×Lacaune=14; KKCF samples: *n* = 111; Merino=43; Romane=14; X-ILFrance=25; X-Merino=6; X-Romane=11; X-Suffolk=12), and heterogeneity of variances was significant (*P* < 0.01), a pooled SEM could not be presented in tables, and therefore individual SEMs are presented instead.

Graphs were conducted with the software Jamovi v.2.7.2.2. and Spearman’s rank order correlation (ρ) was used to assess relationships between variables using the module of Smolyakov (2025), and a statistical significance was set at *P* < 0.05.

## 3 Results

### 3.1 Carcass weight and pH

Cold carcass weight averaged 12.1 kg (ranging from 8.19 to 17.6 kg) without differences among genetic groups (*P* > 0.05). Their distribution by genetic group is shown in Supplementary Figure S1. Regarding rumen pH, it averaged 5.97, with significant differences among genetic groups (Supplementary Figure S2; *P* < 0.05). The X-Suffolk showed the highest mean pH values of 6.34, whereas Merino×Lacaune showed the lowest (5.83) and less variable pH distributions. Purebred Merino displayed the largest range of pH values and an average of 5.88, indicating greater inter-individual variability within this genetic group. Intermediate pH values were observed in X-Romane (6.08), X-ILFrance (5.87), and Romane (6.04).

### 3.2 Rumen volatile fatty acids

Volatile fatty acid concentrations and proportions in the rumen contents of lambs are presented in Table 1. Total VFA concentration varied between genetic groups (*P* < 0.001), with crossbred lambs, except for Merino×Lacaune, generally showing higher concentrations than purebred animals, with the highest values observed in X-Suffolk (90.5 mmol/L) and X-Merino lambs (89.2 mmol/L). In contrast, Romane lambs showed the lowest total VFA concentration (23.3 mmol/L), while Merino lambs presented intermediate values (34.6 mmol/L). Significant effects among groups were also observed for individual VFA. The molar proportion of acetic acid (2:0) differed among genetic groups (*P* < 0.001), ranging from 55.6 % in X-Suffolk lambs to 60.4 % in Romane lambs. Propionic acid (3:0) also varied (*P* < 0.001), with Merino, X-ILFrance and Merino×Lacaune lambs presenting higher proportions (29.3 %, 29.3 % and 30.1 %, respectively) compared with Romane lambs. The proportion of isobutyric acid (i-4:0) was significantly affected by genetic group (*P* < 0.001), with Romane lambs showing the highest value and X-Merino lambs the lowest (1.96 %). Similarly, butyric acid (4:0) proportions differed significantly across groups (*P* < 0.001), with the highest values observed in X-Merino (8.76 %) and the lowest in Merino×Lacaune lambs (5.78 %). Isovaleric acid (i-5:0) also varied among genetic groups (*P* = 0.002), with X-Merino lambs showing the lowest proportions (1.67 %). The valeric acid (5:0), also varied among genetic groups, but its proportion was lower than 1.5 %.

**Table 1:** Volatile fatty acid (VFA) concentration (mmol/L) and composition (% total VFA) in the rumen contents of lambs from different genetic groups (values are mean ± standard error of the mean).

AGV	Merino	Romane	X-ILFrance^1^	M×Lacaune^2^	X-Merino^1^	X-Romane^1^	X-Suffolk^1^	*P*-value
Total VFA	34.6^c^ ± 1.55	23.3^d^ ± 2.09	48.9^b^ ± 3.39	32.2^cd^ ± 10.49	89.2^a^ ± 8.50	54.4^b^ ± 6.21	90.5^a^ ± 9.81	<0.001
2:0	56.7^b^ ± 0.43	60.4^ab^ ± 1.34	56.2^b^ ± 0.66	56.3^b^ ± 0.68	59.5^a^ ± 0.76	60.2^a^ ± 0.82	55.6^ab^ ± 1.58	<0.001
3:0	29.3^a^ ± 0.43	23.9^b^ ± 1.28	29.3^a^ ± 0.62	30.1^a^ ± 0.99	26.9^ab^ ± 0.68	25.1^b^ ± 0.76	28.0^ab^ ± 1.05	<0.001
i-4:0	3.29^b^ ± 0.158	5.05^a^ ± 0.463	3.55^ab^ ± 0.252	3.57^ab^ ± 0.329	1.96^c^ ± 0.204	2.77^bc^ ± 0.293	4.66^ab^ ± 0.705	<0.001
4:0	7.17^bc^ ± 0.168	6.24^cd^ ± 0.288	7.53^ab^ ± 0.243	5.78^d^ ± 0.368	8.76^a^ ± 0.418	8.09^ab^ ± 0.396	8.00^ab^ ± 0.360	<0.001
i-5:0	2.75^a^ ± 0.126	3.64^a^ ± 0.316	2.61^a^ ± 0.152	2.93^a^ ± 0.292	1.67^b^ ± 0.175	2.94^a^ ± 0.285	2.46^ab^ ± 0.310	<0.001
5:0	0.86^b^ ± 0.035	0.64^b^ ± 0.066	0.84^b^ ± 0.042	1.48^a^ ± 0.108	1.29^a^ ± 0.094	0.90^b^ ± 0.074	1.28^ab^ ± 0.234	<0.001
2:0/3:0	2.07^bc^± 0.046	2.95^a^ ± 0.225	2.11^bc^ ± 0.087	1.91^c^ ± 0.079	2.31^ab^ ± 0.086	2.67^a^ ± 0.132	2.18^abc^ ± 0.133	<0.001

Letters in the same row with different superscripts are statistically different (*P* < 0.05).

1 Ewes that do not have a clear breed affiliation were crossed with purebred rams. ^2^Lacaune ewes crossed with Merino rams.

### 3.3 Rumen dimethyl acetals

The composition of DMA in the rumen contents of lambs differed significantly among genetic groups (Table 2). Total DMA concentration was highest in Merino×Lacaune lambs (1310 µg/g DM), but it did not differ from Merino (1184 µg/g DM) and X-Merino lambs (1069 µg/g DM). Lower total DMA concentrations were observed in Romane (858 µg/g DM) but it did not differ from X-Suffolk (885 µg/g DM) and X-Romane lambs (939 µg/g DM). All individual DMA concentrations, except i-15:0, showed significant differences among genetic groups (*P* < 0.001). The Merino and/or Merino×Lacaune generally had the highest concentrations of i-13:0, 13:0, i-14:0, 14:0, a-15:0, 15:0, and 18:0 DMA. The X-Merino also presented the highest 16:0 and 18:1 concentration. The lowest concentration of individual DMA are often in X-Romane, Romane and X-Suffolk.

**Table 2:** Dimethyl acetal (DMA) composition (µg/g DM) in the rumen contents of lambs from different genetic groups (values are mean ± standard error of the mean).

DMA	Merino	Romane	X-ILFrance	M×Lacaune	X-Merino	X-Romane	X-Suffolk	*P*-value
Total DMA	1184^a^ ± 21.1	858^c^ ± 51.2	1032^b^ ± 28.7	1310^a^ ± 89.3	1069^ab^ ± 47.8	938^bc^ ± 37.3	885^bc^ ± 52.1	<0.001
i-13:0	14.4^a^ ± 1.55	9.7^ab^ ± 2.23	5.3^b^ ± 1.22	15.5^ab^ ± 5.00	4.8^b^ ± 1.70	4.3^b^ ± 1.37	6.8^ab^ ± 2.00	<0.001
13:0	29.0^a^ ± 1.60	16.4^b^ ± 2.56	26.9^a^ ± 1.97	38.4^ab^ ± 9.43	23.4^ab^ ± 4.64	16.1^b^ ± 1.52	19.9^ab^ ± 3.53	<0.001
i-14:0	158^a^ ± 4.5	103^b^ ± 8.9	153^a^ ± 6.8	215^a^ ± 25.6	137^ab^ ± 11.4	115^b^ ± 7.3	106^b^ ± 7.6	<0.001
14:0	150^ab^ ± 3.8	107^c^ ± 9.1	144^ab^ ± 5.1	182^a^ ± 15.9	128^bc^ ± 8.5	111^c^ ± 6.6	110^c^ ± 9.3	<0.001
i-15:0	6.9 ± 1.24	7.4 ± 2.21	3.6 ± 0.76	5.7 ± 4.05	6.3 ± 1.66	4.6 ± 0.85	3.5 ± 1.36	0.231
a-15:0	282^ab^ ± 7.7	204^cd^ ± 15.0	251^abc^ ± 10.3	358^a^ ± 37.8	231^bcd^ ± 16.9	209^cd^ ± 9.7	182^d^ ± 13.4	<0.001
15:0	98.9^a^ ± 2.52	67.2^cd^ ± 5.27	80.2^b^ ± 3.59	102^a^ ± 19.6	77.3^bc^ ± 5.07	68.4^bc^ ± 2.70	57.1^c^ ± 5.23	<0.001
16:0	345^ab^ ± 7.4	263^c^ ± 15.2	290^c^ ± 7.1	295^bc^ ± 18.5	368^a^ ± 13.4	319^abc^ ± 12.8	322^abc^ ± 28.7	<0.001
18:0	42.9^a^ ± 2.11	32.2^abc^ ± 4.25	31.4^bc^ ± 1.87	52.1^abc^ ± 21.4	29.3^bc^ ± 2.53	33.7^b^ ± 1.69	21.3^c^ ± 3.17	<0.001
18:1	57.4^ab^ ± 1.73	46.8^bc^ ± 3.52	47.3^c^ ± 2.33	48.9^abc^ ± 12.4	65.5^a^ ± 4.32	56.4^abc^ ± 2.57	57.1^abc^ ± 5.61	0.001

Letters in the same row with different superscripts are statistically different (*P* < 0.05).

### 3.4 Rumen fatty acids

All individual FA and FA classes of rumen contents (mg/100 g DM) presented in Table 3 differed (*P* < 0.01) among lamb genetic groups. Saturated linear chain FA (linear-SFA) represented the predominant FA class across all groups. Among these, stearic acid (18:0) was the most abundant, with the highest concentrations observed in Merino (1691 mg/100 g DM), but it did not differ from Merino×Lacaune and X-Merino, and the lowest concentration was found in X-Suffolk lambs (1218 mg/100 g DM). Palmitic acid (16:0) was the second most abundant Linear-SFA with concentrations ranging from 486 mg/100 g DM in Romane lambs to 655 mg/100 g DM in Merino×Lacaune lambs. Besides these two major FA, all the other linear-SFA are present in much lower concentration, but with 15:0 (ranging from 46 mg/100 g DM in X-Suffolk to 83 mg/100 g DM in Merino×Lacaune) and 14:0 (ranging from 36 mg/100 g DM in Romane to 57 mg/100 g DM in Merino and Merino×Lacaune) as the third and fourth major linear-SFA.

**Table 3:** Fatty acid (FA) composition (mg/100 g DM) in the rumen contents of lambs from different genetic groups (values are mean ± standard error of the mean).

FA	Merino	Romane	X-ILFrance	M×Lacaune	X-Merino	X-Romane	X-Suffolk	*P*-value
Linear-SFA	2525^a^ ± 48	2095^b^ ± 116	2202^b^ ± 65	2333^ab^ ± 203	2335^ab^ ± 108	2143^b^ ± 85	1922^b^ ± 118	<0.001
12:0	8.0^a^ ± 0.26	4.2^c^ ± 0.63	7.4^ab^ ± 0.36	7.4^abc^ ± 1.11	9.0^a^ ± 0.59	6.3^bc^ ± 0.46	6.8^abc^ ± 0.65	<0.001
13:0	4.2^a^ ± 0.17	2.0^b^ ± 0.29	3.6^a^ ± 0.21	4.8^ab^ ± 0.10	3.8^a^ ± 0.32	2.7^b^ ± 0.16	2.5^b^ ± 0.30	<0.001
14:0	57^a^ ± 1.8	36^b^ ± 3.1	53^a^ ± 2.6	57^a^ ± 4.5	52^a^ ± 2.6	40^b^ ± 1.9	40^b^ ± 2.6	<0.001
15:0	72^a^ ± 1.6	54^bc^ ± 3.8	61^b^ ± 2.1	83^a^ ± 6.6	54^bc^ ± 3.5	51^bc^ ± 2.7	46^c^ ± 3.8	<0.001
16:0	585^a^ ± 11.7	486^b^ ± 28.5	574^ab^ ± 16.0	655^ab^ ± 49.7	640^a^ ± 26.6	564^ab^ ± 20.8	541^ab^ ± 29.0	0.003
17:0	29^a^ ± 0.6	25^b^ ± 1.0	23^bc^ ± 0.9	25^abc^ ± 2.0	22^bc^ ± 0.8	22^bc^ ± 0.7	20^c^ ± 1.2	<0.001
18:0	1691^a^ ± 40	1403^b^ ± 75	1427^b^ ± 46	1440^ab^ ± 150	1498^ab^ ± 68	1382^b^ ± 47	1218^b^ ± 73	<0.001
20:0	27^a^ ± 0.5	23^b^ ± 0.8	20^bc^ ± 0.5	21^bc^ ± 1.7	20^bc^ ± 0.8	21^bc^ ± 0.5	18^c^ ± 0.9	<0.001
21:0	1.8^a^ ± 0.14	1.3^bc^ ± 0.16	0.9^c^ ± 0.09	1.0^abc^ ± 0.38	1.2^bc^ ± 0.12	1.4^b^ ± 0.06	1.1^bc^ ± 0.16	<0.001
22:0	14^a^ ± 0.4	11^bc^ ± 0.9	10^c^ ± 0.3	10^c^ ± 0.8	13^ab^ ± 0.6	13^a^ ± 0.4	14^a^ ± 0.7	<0.001
23:0	7.1^a^ ± 0.48	2.5^b^ ± 0.54	2.0^b^ ± 0.17	1.6^b^ ± 0.57	2.4^b^ ± 0.30	2.5^b^ ± 0.19	1.7^b^ ± 0.28	<0.001
24:0	14^abc^ ± 0.5	17^a^ ± 1.7	13^bc^ ± 0.6	11^c^ ± 1.0	12^c^ ± 0.5	15^ab^ ± 0.6	12^c^ ± 0.7	0.010
26:0	11^b^ ± 0.8	18^a^ ± 1.8	6.8^c^ ± 0.39	5.2^c^ ± 1.70	5.9^c^ ± 0.46	12^b^ ± 1.1	6.0^c^ ± 0.64	<0.001
28:0	4.2^b^ ± 0.62	14^a^ ± 2.7	1.2^c^ ± 0.17	0.4^d^ ± 0.23	0.3^d^ ± 0.12	9.8^ab^ ± 1.9	0.4^d^ ± 1.56	<0.001
BCFA	165^a^ ± 3.5	131^b^ ± 6.9	125^b^ ± 3.8	134^b^ ± 9.2	124^b^ ± 4.9	124^b^ ± 3.7	99^c^ ± 5.2	<0.001
i-13:0	2.0^a^ ± 0.13	1.2^b^ ± 0.19	1.3^b^ ± 0.12	1.6^ab^ ± 0.50	1.4^ab^ ± 0.22	1.3^b^ ± 0.12	0.8^b^ ± 0.14	<0.001
i-14:0	13^a^ ± 0.3	8^d^ ± 0.5	11^b^ ± 0.4	15^ab^ ± 1.7	11^abc^ ± 0.7	10^bcd^ ± 0.4	9^cd^ ± 0.5	<0.001
i-15:0	33^a^ ± 0.9	32^a^ ± 2.1	25^b^ ± 0.9	27^abc^ ± 2.3	22^bc^ ± 1.0	23^bc^ ± 1.0	20^c^ ± 1.5	<0.001
a-15:0	64^a^ ± 1.3	45^bc^ ± 2.8	54^b^ ± 2.2	51^bc^ ± 3.1	51^b^ ± 2.1	48^bc^ ± 1.5	41^c^ ± 2.3	<0.001
i-16:0	17^a^ ± 0.4	13^b^ ± 0.8	13^b^ ± 0.4	15^ab^ ± 1.8	12^b^ ± 0.7	13^b^ ± 0.5	12^b^ ± 1.0	<0.001
i-17:0	17^a^ ± 2.4	13^a^ ± 1.1	9.6^bc^ ± 0.4	10^abc^ ± 1.1	11^abc^ ± 0.7	11^ab^ ± 0.5	8.5^c^ ± 0.73	<0.001
a-17:0	14^a^ ± 0.5	16^a^ ± 1.3	9.4^b^ ± 0.50	11^ab^ ± 1.7	13^a^ ± 0.9	15^a^ ± 0.9	7.0^b^ ± 0.82	<0.001
i-18:0	2.9^a^ ± 0.13	2.3^abc^ ± 0.27	1.8^bc^ ± 0.15	3.6^abc^ ± 1.69	2.1^abc^ ± 0.31	2.3^b^ ± 0.14	1.3^c^ ± 0.25	<0.001
MUFA	756^a^ ± 20.1	528^b^ ± 35.8	664^ab^ ± 29.2	640^ab^ ± 43.5	661^ab^ ± 29.0	596^b^ ± 23.1	662^ab^ ± 38.5	<0.001
16:1	4.8^a^ ± 0.28	2.0^c^ ± 0.27	3.9^ab^ ± 0.37	3.8^ab^ ± 0.59	3.2^b^ ± 0.30	2.1^c^ ± 0.15	2.1^c^ ± 0.27	<0.001
18:1^1^	747^a^ ± 20.1	522^b^ ± 35.4	656^ab^ ± 28.9	633^ab^ ± 43.1	654^ab^ ± 28.8	590^b^ ± 22.9	656^ab^ ± 38.2	<0.001
20:1	5.0^a^ ± 0.24	4.6^ab^ ± 0.41	3.2^c^ ± 0.17	2.8^bc^ ± 0.55	3.7^bc^ ± 0.30	4.2^ab^ ± 0.23	3.5^abc^ ± 0.43	<0.001
PUFA	86^a^ ± 2.3	92^a^ ± 5.5	62^bc^ ± 3.1	52^c^ ± 9.6	87^ab^ ± 5.1	94^a^ ± 4.0	82^ab^ ± 5.6	<0.001
*t*11*c*15/*t*10*c*15-18:2	4.9^a^ ± 0.25	3.7^ab^ ± 0.47	3.1^b^ ± 0.20	2.7^b^ ± 0.63	3.2^b^ ± 0.37	3.6^b^ ± 0.27	5.1^ab^ ± 1.4	0.001
18:2n-6	65^a^ ± 1.3	73^a^ ± 4.1	49^bc^ ± 1.9	42^c^ ± 3.1	70^ab^ ± 7.2	76^a^ ± 4.8	66^ab^ ± 5.3	<0.001
18:3n-3	6.8^b^ ± 0.30	9.0^a^ ± 0.72	5.4^cd^ ± 0.41	3.0^d^ ± 1.26	7.2^abc^ ± 0.67	8.9^a^ ± 0.53	6.6^abc^ ± 0.73	<0.001
*c*9*t*11-CLA	3.6^a^ ± 0.21	1.5^c^ ± 0.27	2.5^bc^ ± 0.23	2.4^abc^ ± 1.1	3.5^ab^ ± 0.35	2.9^abc^ ± 0.54	2.2^abc^ ± 0.48	0.003
*t*10*c*12-CLA	2.2^a^ ± 0.16	1.1^bc^ ± 0.24	0.5^c^ ± 0.07	0.4^c^ ± 0.19	1.5^ab^ ± 0.25	1.2^b^ ± 0.13	1.0^bc^ ± 0.18	<0.001
*c*9*t*11*c*15-18:3	3.7^a^ ± 0.34	3.9^ab^ ± 0.13	1.3^b^ ± 0.16	1.2^ab^ ± 0.88	1.6^b^ ± 0.18	1.6^b^ ± 0.11	1.1^b^ ± 0.17	<0.001
Others	47^ab^ ± 1.7	34^c^ ± 3.1	57^a^ ± 4.3	72^a^ ± 8.5	50^ab^ ± 3.2	40^bc^ ± 2.7	48^abc^ ± 5.0	<0.001
Total FA	3579^a^ ± 64	2884^b^ ± 127	3110^b^ ± 88	3217^ab^ ± 250	3255^ab^ ± 104	2998^b^ ± 73	2811^b^ ± 118	<0.001

Letters in the same row with different superscripts are statistically different (*P* < 0.05).

1 Sum of all 18:1 *cis* and *trans* isomers.

Branched-chain FA (BCFA) also contributed substantially to total FA content, and all differed among genetic groups (*P* < 0.001). Anteiso-15:0 (a-15:0) and iso-15:0 (i-15:0) were the predominant BCFA. Merino lambs showed the highest a-15:0 concentration (64 mg/100 g DM) and i-15:0 concentration (33 mg/100 g DM), and X-Suffolk lambs the lowest concentration of a-15:0 (41 mg/100 g DM) and i-15:0 (20 mg/100 g DM). The total monounsaturated FA (MUFA) varied significantly among genetic groups (*P* < 0.001), but the total 18:1 isomers were the predominant MUFA across all groups, with concentrations ranging from 522 mg/100 g DM in Romane lambs to 747 mg/100 g DM in Merino lambs. The detailed composition of 18:1 isomers is presented in Table 4.

**Table 4:** C18 fatty acids (% of total C18) in the rumen contents of lambs from different genetic groups (values are mean ± standard error of the mean).

FA	Merino	Romane	X-ILFrance	M×Lacaune	X-Merino	X-Romane	X-Suffolk	*P*-value
18:0	65^a^ ± 0.7	69^a^ ± 1.6	65^ab^ ± 0.9	64^ab^ ± 2.9	65^ab^ ± 1.5	65^ab^ ± 1.2	60^b^ ± 1.7	0.029
*trans*-18:1								
*t*6/*t*7/*t*8-	1.2^a^ ± 0.03	1.0^d^ ± 0.06	1.1^bd^ ± 0.03	1.3^ab^ ± 0.09	1.3^ac^ ± 0.06	1.1^bcd^ ± 0.04	1.4^ab^ ± 0.10	<0.001
*t*9-	0.57^a^ ± 0.017	0.43^b^ ± 0.032	0.53^ab^ ± 0.039	0.70^ab^ ± 0.095	0.60^a^ ± 0.032	0.57^ab^ ± 0.053	0.60^ab^ ± 0.049	0.004
*t*10-	14^a^ ± 0.5	9.9^b^ ± 1.2	15^a^ ± 0.7	16^ab^ ± 2.0	14^ab^ ± 1.1	12^ab^ ± 0.9	13^ab^ ± 1.2	0.005
*t*11-	3.1^b^ ± 0.17	4.0^b^ ± 0.48	3.0^b^ ± 0.27	1.6^c^ ± 0.17	2.3^bc^ ± 0.29	3.4^b^ ± 0.29	7.4^a^ ± 0.98	<0.001
*t*12-	0.90^a^ ± 0.038	0.68^b^ ± 0.045	0.77^ab^ ± 0.025	0.77^ab^ ± 0.063	0.83^ab^ ± 0.035	0.80^ab^ ± 0.030	0.94^ab^ ± 0.094	0.010
*t*15-	0.71^a^ ± 0.017	0.67^ab^ ± 0.033	0.60^b^ ± 0.019	0.66^ab^ ± 0.088	0.63^ab^ ± 0.028	0.70^a^ ± 0.022	0.63^ab^ ± 0.031	0.002
*t*16-1	0.48^ab^ ± 0.015	0.51^abc^ ± 0.036	0.40^c^ ± 0.020	0.30^bc^ ± 0.063	0.41^bc^ ± 0.034	0.50^ab^ ± 0.026	0.56^a^ ± 0.037	<0.001
*cis-18:1*								
*c*9-2	6.7^b^ ± 0.11	6.6^ab^ ± 0.19	6.9^ab^ ± 0.16	7.1^ab^ ± 0.35	7.2^ab^ ± 0.22	7.5^a^ ± 0.23	7.5^ab^ ± 0.26	0.007
*c*11-	0.98^a^ ± 0.025	0.75^c^ ± 0.050	0.90^abc^ ± 0.026	1.0^abc^ ± 0.08	0.98^ab^ ± 0.042	0.85^bc^ ± 0.035	0.85^abc^ ± 0.063	<0.001
*c*12-	0.51^a^ ± 0.018	0.58^a^ ± 0.055	0.31^c^ ± 0.016	0.25^c^ ± 0.044	0.39^bc^ ± 0.030	0.50^ab^ ± 0.039	0.43^ab^ ± 0.037	<0.001
*c*13-	0.07^b^ ± 0.007	0.03^c^ ± 0.006	0.06^bc^ ± 0.010	0.26^a^ ± 0.038	0.13^b^ ± 0.023	0.03^c^ ± 0.003	0.07^bc^ ± 0.016	<0.001
*c*15-	0.25 ± 0.013	0.24 ± 0.025	0.25 ± 0.014	0.23 ± 0.037	0.22 ± 0.024	0.28 ± 0.016	0.26 ± 0.036	0.541
*c*16-	0.10^a^ ± 0.007	0.05^b^ ± 0.008	0.09^ab^ ± 0.035	0.07^ab^ ± 0.026	0.07^ab^ ± 0.009	0.06^b^ ± 0.004	0.07^ab^ ± 0.011	<0.001
18:2								
*t*11*c*15/*t*10*c*15-	0.19^a^ ± 0.009	0.19^ab^ ± 0.023	0.15^bc^ ± 0.011	0.12^c^ ± 0.023	0.15^bc^ ± 0.018	0.18^ab^ ± 0.015	0.28^abc^ ± 0.084	0.020
*c*9*c*12- (n-6)	2.7^bc^ ± 0.09	3.7^a^ ± 0.21	2.4^cd^ ± 0.10	2.0^d^ ± 0.15	3.1^abc^ ± 0.29	3.7^a^ ± 0.22	3.3^ab^ ± 0.22	<0.001
*c*9*t*11-CLA	0.13^a^ ± 0.007	0.07^b^ ± 0.011	0.10^ab^ ± 0.009	0.10^ab^ ± 0.039	0.15^a^ ± 0.013	0.13^ab^ ± 0.024	0.11^ab^ ± 0.020	<0.001
*t*10*c*12-CLA	0.09^a^ ± 0.007	0.05^bc^ ± 0.009	0.02^c^ ± 0.003	0.02^c^ ± 0.010	0.06^ab^ ± 0.010	0.06^b^ ± 0.006	0.05^bc^ ± 0.008	<0.001
18:3								
*c*9*c*12*c*15- (n-3)	0.29^c^ ± 0.014	0.48^a^ ± 0.035	0.28^c^ ± 0.021	0.13^d^ ± 0.028	0.32^abc^ ± 0.040	0.44^ab^ ± 0.032	0.32^bc^ ± 0.037	<0.001
*c*9*t*11*c*15-	0.15^a^ ± 0.014	0.16^ab^ ± 0.046	0.06^b^ ± 0.009	0.08^ab^ ± 0.066	0.07^b^ ± 0.008	0.07^b^ ± 0.005	0.05^b^ ± 0.008	<0.001
oxo-18:0								
10-oxo^3^	1.5^cd^ ± 0.06	1.2^d^ ± 0.11	2.3^a^ ± 0.16	3.2^a^ ± 0.45	2.1^ab^ ± 0.16	1.6^bcd^ ± 0.12	2.2^abc^ ± 0.27	<0.001
13-oxo	0.22^a^ ± 0.016	0.24^a^ ± 0.034	0.08^b^ ± 0.008	0.17^ab^ ± 0.047	0.07^b^ ± 0.009	0.09^b^ ± 0.011	0.09^b^ ± 0.010	<0.001
Ratio and sums								
*t*10-shift^4^	7.9^ab^ ± 0.37	4.8^c^ ± 0.78	9.1^a^ ± 0.64	10.5^abc^ ± 1.79	10.9^a^ ± 1.52	7.7^abc^ ± 1.06	5.1^bc^ ± 1.01	<0.001
BI^5^	24^ab^ ± 0.6	20^b^ ± 1.4	25^ab^ ± 0.8	26^ab^ ± 2.5	24^ab^ ± 1.4	22^b^ ± 1.1	28^a^ ± 1.5	0.003

Letters in the same row with different superscripts are statistically different (*P* < 0.05).

1 Might coelute with *c*14-18:1 as minor isomer.

2 Might coelute with *t*13-18:1 and *t*14-18:1.

3 Might coelute with other oxo-18:0, as minor isomers.

4 Ratio between *t*10-18:1 and *t*11-18:1.

5 Biohydrogenation intermediates, sum of all C18 fatty acids excluding 18:0, *c*9-18:1, *c*11-18:1, 18:2n-6 and 18:3n-3.

Polyunsaturated FA (PUFA) were present at lower concentrations than Linear-SFA, MUFA and even BCFA, ranging from 52 mg/100 g DM in Merino×Lacaune to 94 mg/100 g DM in X-Romane. Linoleic acid (18:2 n-6) was the predominant polyunsaturated FA (PUFA), with higher concentrations observed in X-Romane lambs (76 mg/100 g DM) and lowest in Merino×Lacaune (42 mg/100 g DM). α-Linolenic acid (18:3 n-3) differed among genetic groups following the same pattern described for 18:2 n-6. Rumenic acid (*cis*-9*trans*-11 CLA) was the major PUFA BH intermediate with the highest concentration observed in Merino lambs (≈ 3.6 mg/100 g DM), but it only differed from Romane and X-ILFrance.

### 3.5 Relative proportions of C18 fatty acids and trans-10 shift in rumen

The relative composition of C18 FA in the rumen contents of lambs is presented in Table 4. Stearic acid (18:0) was the predominant C18 FA, accounting for approximately 60–69 % of total C18 FA, with only a tendency to vary by genetic group (*P* = 0.029). In contrast, several 18:1 isomers differed among groups (*P* < 0.01), the exception was the *c*15-18:1 (*P* = 0.541).

**Fig. 1:**
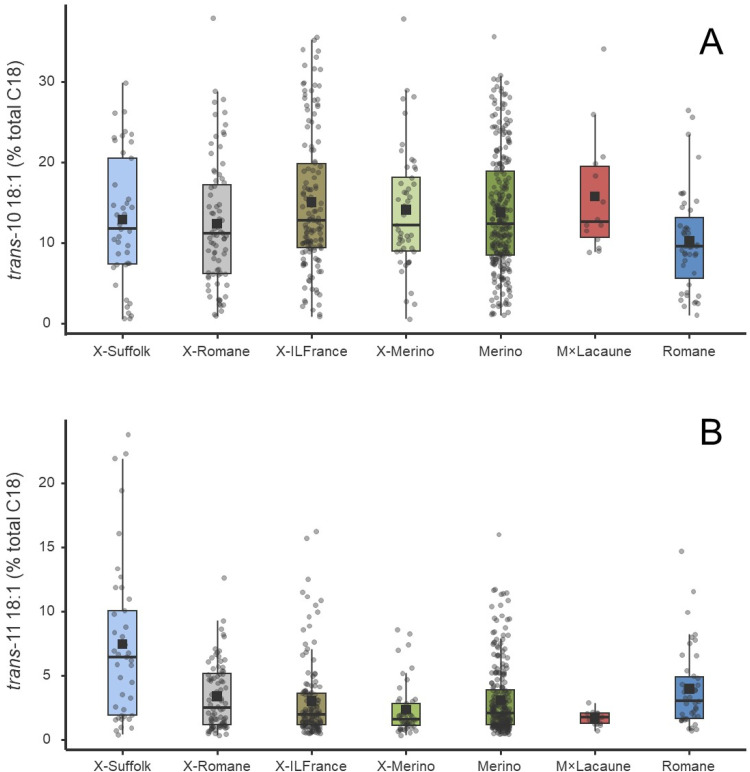
Distribution of *trans*-10 18:1 (panel A) and *trans*-11 18:1 (panel B) in proportion of total C18 fatty acids in rumen content across genetic groups. Boxplots represent the median (horizontal line), interquartile range (box), mean (square dot) and minimum–maximum values. Individual observations are shown as jittered points. Genetic group significantly affected *trans*-11 18:1 concentration (*P* < 0.01).

Among individual *trans*-18:1 isomers, *t*10-18:1 was the most abundant, comprising, on average, circa 10 % of total C18 FA, but it varied between genetic groups (*P* = 0.005). Despite these differences, the dispersion of proportions was quite large, as shown in Fig. 1A, and consequently, only Merino (14 %) and X-ILFrance (15 %) differed from Romane, which presented the lowest average proportion (i.e. 9.9 % total C18 FA). The *t*11-18:1 comprised, on average, 3.6 % of total C18 FA, but it also varied between groups (*P* < 0.001). However, the dispersion of the values is not as large as for *t*10-18:1 (Fig. 1B). Regarding genetic groups, the Merino×lacaune had the lowest proportion of *t*11-18:1 (1.6 % of total C18 FA), whereas the X-Suffolk had the highest (7.4 % of total C18). The descriptive statistics of *t*10-18:1 and *t*11-18:1 ( % of total C18 FA) in rumen content according to genetic groups are presented in the supplementary Table S3.

**Fig. 2:**
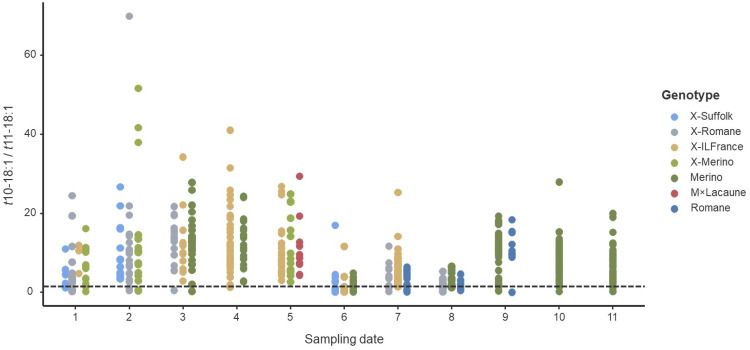
Variation of the *trans*-10 shift ratio (*trans*-10 18:1 / *trans*-11 18:1) across sampling dates (1-11) and genetic group. Each point represents an individual animal, and colors denote genetic groups. The dashed horizontal line indicates a *trans*-10 shift threshold value of 1. (For interpretation of the references to color in this figure legend, the reader is referred to the web version of this article.).

The *t*10-18:1/*t*11-18:1 ratio varied among genetic groups (*P* < 0.001) and differed significantly between sampling dates (Fig. 2). Of the 611 samples analysed for FA in rumen content, only 10.6 % (65 samples) showed a *t*10-shift ratio <1, and these samples were fairly distributed across genetic groups and sampling dates. The 65 animals were distributed across genetic groups as follows: 15 X-Suffolk, 18 Merino, 11 X-Romane, 9 X-ILFrance, 8 Romane and 4 X-Merino. Thus, about 37 % of the total X-Suffolk lambs, 19 % of the Romane lambs, and 14 % of the X-Romane lambs had the *t*10-shift ratio <1, whereas only 7 % of the total Merino lambs, 8 % of X-Merino lambs and 7 % of X-ILFrance lambs shared the same phenotype. Within *cis*-MUFA, the oleic acid (*cis*-9 18:1) was the predominant C18 FA in the rumen content and showed significant genetic group-related differences (*P* = 0.007), with higher proportions observed in X-Romane lambs (7.5 % of total C18 FA) compared with Merino lambs (6.7 % total C18 FA).

Linoleic acid (18:2 n-6) differed significantly among genetic groups (*P* < 0.001), with the highest proportions observed in Romane purebred and crossed (3.7 % total C18) and the lowest in Merino×Lacaune lambs (2.0 % of total C18). α-Linolenic acid (18:3 n-3) also varied significantly (*P* < 0.001), with the highest proportion detected in Romane lambs (0.48 % of total C18). Regarding the 18:2 and 18:3 BH intermediates, all varied among groups (*P* < 0.001), with the pair *t*11*c*15/*t*10*c*15-18:2 presenting the highest values in X-Suffolk (0.28 % of total C18). Merino and X-Merino lambs showed the highest values of *c*9*t*11-CLA (0.13 % and 0.15 % total C18), Merino the highest values of *t*10*c*12-CLA (0.09 % total C18) and Merino and Romane the highest values of *c*9*t*11*c*15-18:3 (0.15 % and 0.16 % total C18, respectively). In line with these results, the total BH intermediates (BI) differed significantly among genetic groups (*P* = 0.003), with X-Suffolk lambs presenting the highest proportion (28 % of total C18 FA) and Romane and X-Romane lambs the lowest (20 % and 22 % of total C18 FA, respectively).

### 3.6 Correlations between C18 fatty acids and pH in rumen

**Fig. 3:**
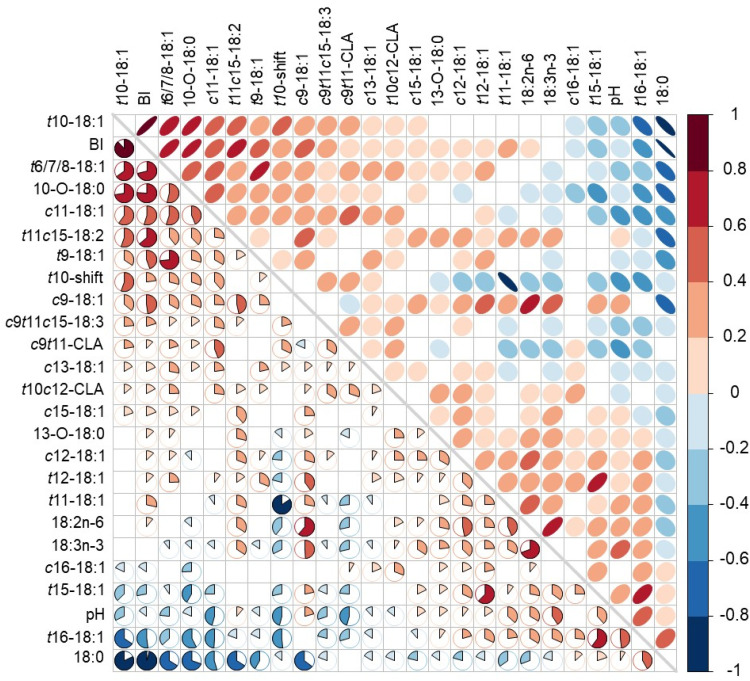
Spearman’s rank correlation matrix among C18 fatty acids, biohydrogenation intermediates, indices (BI, *t*10-shift), and rumen pH. Color intensity and ellipse orientation indicate the strength and direction of correlations (red = positive; blue = negative). Only significant correlations (*P* < 0.05) are shown as filled ellipses. Variables are listed according to the strength of the positive to negative correlations between *t*10-18:1 and other variables. (For interpretation of the references to color in this figure legend, the reader is referred to the web version of this article.).

Spearman’s rank correlation coefficient (ρ) analysis revealed clear relationships among BH intermediates (Fig. 3). The *t*10-18:1, 10-oxo-18:0, the *t*10-shift ratio, and the BH intermediates were positively correlated with each other (*P* < 0.001). In contrast, these variables were negatively correlated with 18:0, pH and *t*16-18:1(*P* < 0.001). Surprisingly, *t*10-18:1 was not significantly correlated with *t*11-18:1 (*P* > 0.05), however, the *t*10-shift and *c*9*t*11-CLA were negatively correlated with *t*11-18:1. The 18:2n-6 and 18:3n-3 were also negatively correlated with *c*9*t*11-CLA and 18:0. This latter FA was negatively correlated with most of the C18 FA, except with *t*15-18:1, *t*16-18:1 and *c*16-18:1, but was positively correlated with pH (*P* < 0.05). The complete correlation matrix with Spearman’s rank correlation coefficients and significances is presented in Supplementary Table S4.

**Fig. 4:**
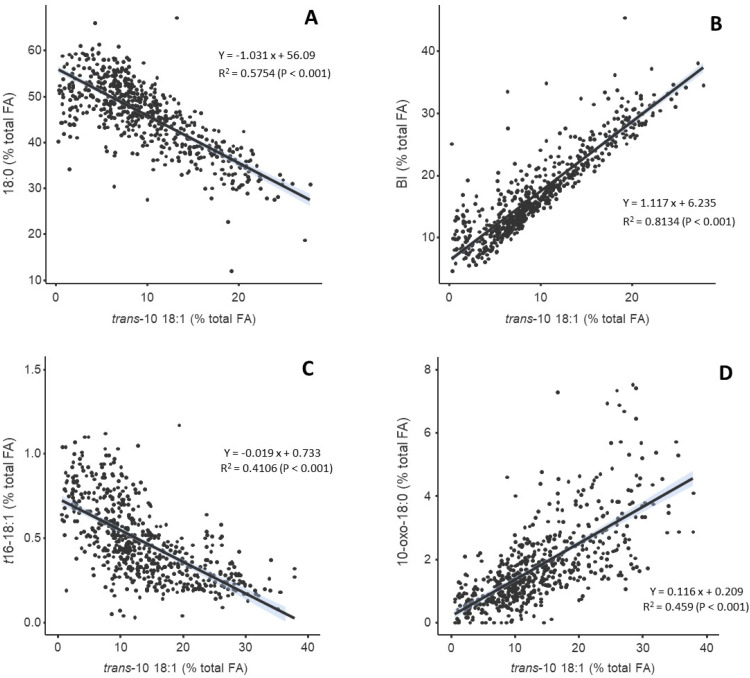
Relationships between *trans*-10 18:1 (% of total fatty acids) in the rumen content of lambs with: (A) 18:0, (B) the sum of biohydrogenation intermediates (BI), (C) *t*16-18:1, and (D) 10-oxo-18:0 (% total FA) in the rumen content. Each point represents an individual sample. Solid lines indicate linear regression fits with 95 % confidence intervals.

In addition, Fig. 4 shows graphic representations of the correlations between *t*10-18:1 with 18:0 (Panel A), total BH intermediates (Panel B), *t*16-18:1 (Panel C) and 10-oxo-18:0 (Panel D). The figure shows that the *t*10-18:1 was negatively associated with 18:0 content, indicating that increasing accumulation of *t*10-18:1 coincided with reduced formation of the final BH product (18:0), and was also negatively correlated with *t*16-18:1 (R^2^ = 0.4106, *P* < 0.001). Conversely, *t*10-18:1 showed a strong positive relationship with the sum of BI (R^2^ = 0.8134, *P* < 0.001) and 10-oxo-18:0 (R^2^ = 0.459, *P* < 0.001). The Supplementary Figure S3 also shows that the relationship between *t*10-18:1 and 18:0 is independent of both genetic group and sampling date.

### 3.7 Fatty acid composition of adipose tissue

The FA profile of adipose tissue among genetic groups is presented in Table 5. The Linear-SFA accounted for more than half of total FA (51.3–55.0 %) and collectively did not differ significantly among groups (*P* = 0.247). Nevertheless, several individual Linear-SFA showed breed-related differences, including the 10:0, 12:0, 14:0, and 17:0 (*P* < 0.05), with X-Suffolk generally presenting the highest proportions, except for 17:0, which showed the lowest proportion on this breed.

**Table 5:** Fatty acid composition (mg/g of total FA) in the kidney knob channel fat of lambs from different genetic groups (values are mean ± standard error of the mean).

FA	Merino	Romane	X-ILFrance	X-Merino	X-Romane	X-Suffolk	*P*-value
Linear-SFA	530 ± 5.3	513 ± 9.4	532 ± 6.9	538 ± 17.3	550 ± 10.9	532 ± 1.0	0.247
10:0	1.9^bc^ ± 0.13	1.3^c^ ± 0.22	2.2^ab^ ± 0.17	1.7^abc^ ± 0.41	1.9^abc^ ± 0.26	2.6^a^ ± 0.24	0.001
12:0	3.0^bc^ ± 0.32	1.8^c^ ± 0.29	4.1^ab^ ± 0.48	3.0^ab^ ± 0.38	2.5^bc^ ± 0.49	5.0^a^ ± 0.48	0.001
14:0	37.5^b^ ± 2.28	30.0^b^ ± 4.09	43.0^ab^ ± 3.01	39.1^ab^ ± 6.14	33.6^b^ ± 4.51	52.2^a^ ± 4.32	0.005
15:0	5.2 ± 0.17	5.1 ± 0.32	5.5 ± 0.23	5.4 ± 0.48	5.4 ± 0.35	5.5 ± 0.33	0.835
16:0	200 ± 2.6	187 ± 4.7	194 ± 3.5	203 ± 7.1	197 ± 5.2	190 ± 5.0	0.139
17:0	19.3^a^ ± 0.8	21.0^a^ ± 1.32	20.0^a^ ± 1.41	17.8^a^ ± 1.46	20.8^a^ ± 0.97	13.0^b^ ± 0.49	<0.001
18:0	261 ± 5.2	265 ± 9.2	260 ± 6.8	275 ± 13.9	280 ± 10.2	260 ± 9.8	0.516
20:0	2.5 ± 0.12	2.5 ± 0.14	2.5 ± 0.07	2.7 ± 0.23	2.7 ± 0.22	3.3 ± 0.28	0.195
22:0	0.4 ± 0.04	0.4 ± 0.04	0.4 ± 0.03	0.4 ± 0.07	0.5 ± 0.08	0.6 ± 0.07	0.184
24:0	0.03 ± 0.013	0.04 ± 0.024	0.07 ± 0.018	0.04 ± 0.004	0.08 ± 0.027	0.05 ± 0.026	0.552
BCFA	10.7 ± 0.35	11.1 ± 0.63	10.8 ± 0.46	10.8 ± 1.16	10.6 ± 0.73	13.0 ± 0.67	0.076
i-14:0	0.5 ± 0.04	0.6 ± 0.07	0.6 ± 0.05	0.6 ± 0.10	0.5 ± 0.08	0.8 ± 0.07	0.103
i-15:0	1.3 ± 0.07	1.3 ± 0.12	1.2 ± 0.09	1.3 ± 0.18	1.3 ± 0.18	1.6 ± 0.12	0.352
a-15:0	2.2 ± 0.10	2.4 ± 0.17	2.3 ± 0.13	2.3 ± 0.26	2.4 ± 0.19	2.7 ± 0.18	0.395
i-16:0	1.8 ± 0.07	2.0 ± 0.13	1.9 ± 0.10	2.0 ± 0.19	1.8 ± 0.14	2.2 ± 0.14	0.169
i-17:0	3.2^b^ ± 0.12	3.1^b^ ± 0.21	3.2^b^ ± 0.15	3.4^ab^ ± 0.32	3.0^b^ ± 0.23	4.2^a^ ± 0.22	0.004
i-18:0	1.7 ± 0.05	1.9 ± 0.09	1.6 ± 0.06	1.6 ± 0.13	1.7 ± 0.10	1.6 ± 0.09	0.147
MUFA	407 ± 4.7	422 ± 8.3	406 ± 6.1	403 ± 15.4	394 ± 9.7	391 ± 8.8	0.161
*cis*-MUFA	316 ± 4.7	319 ± 8.5	313 ± 6.2	313 ± 12.7	310 ± 9.3	320 ± 8.9	0.964
*c*9-14:1	0.5 ± 0.04	0.5 ± 0.06	0.5 ± 0.05	0.6 ± 0.06	0.6 ± 0.17	0.7 ± 0.08	0.573
*c*7-16:1	4.0^b^ ± 0.13	3.9^b^ ± 0.23	4.3^ab^ ± 0.17	4.1^ab^ ± 0.35	3.9^b^ ± 0.25	5.1^a^ ± 0.24	0.004
*c*9-16:1^1^	13.0 ± 0.32	13.1 ± 1.00	12.8 ± 0.35	13.5 ± 0.89	11.0 ± 1.18	11.6 ± 0.35	0.067
*c*9-17:1	4.5^a^ ± 0.2	4.8^a^ ± 0.31	4.3^a^ ± 0.32	4.0^ab^ ± 0.56	4.8^a^ ± 0.33	2.8^b^ ± 0.11	<0.001
*c*9-18:1^2^	275 ± 4.76	273 ± 8.5	274 ± 6.3	272 ± 12.8	269 ± 9.4	285 ± 9.01	0.868
*c*11-18:1	10.5^b^ ± 0.43	13.3^a^ ± 0.77	9.3^b^ ± 0.56	9.4^abc^ ± 1.15	9.9^b^ ± 0.85	6.2^c^ ± 0.81	<0.001
*c*12-18:1	4.0 ± 0.23	4.4 ± 0.41	2.9 ± 0.30	4.0 ± 0.62	4.1 ± 0.45	3.6 ± 0.44	0.050
*c*13-18:1	0.9^ab^ ± 0.05	1.1^a^ ± 0.08	0.8^b^ ± 0.06	0.8^ab^ ± 0.12	0.8^ab^ ± 0.09	0.7^b^ ± 0.09	0.009
*c*15-18:1	0.4 ± 0.03	0.6 ± 0.12	0.4 ± 0.03	0.4 ± 0.06	0.8 ± 0.19	0.5 ± 0.05	0.085
*c*16-18:1	0.7 ± 0.03	0.6 ± 0.06	0.7 ± 0.04	0.7 ± 0.09	0.7 ± 0.06	0.8 ± 0.06	0.075
*c*9-19:1	1.4 ± 0.08	1.4 ± 0.14	1.3 ± 0.10	1.5 ± 0.21	1.2 ± 0.15	1.0 ± 0.15	0.376
*c*11-19:1	0.4 ± 0.02	0.5 ± 0.04	0.4 ± 0.03	0.4 ± 0.07	0.4 ± 0.05	0.3 ± 0.05	0.232
*c*11-20:1	1.3 ± 0.12	2.4 ± 0.52	1.2 ± 0.05	1.2 ± 0.06	3.2 ± 0.86	1.2 ± 0.05	0.056
*trans*-MUFA	90.9^ab^ ± 5.60	102^a^ ± 4.79	92.4^ab^ ± 5.74	74.9^b^ ± 5.74	85.0^ab^ ± 9.99	71.6^b^ ± 5.73	0.007
*t*6/7/8-18:1	5.5^ab^ ± 0.21	7.2^a^ ± 0.69	5.4^ab^ ± 0.27	5.2^ab^ ± 0.31	6.0^ab^ ± 0.71	4.6^b^ ± 0.31	0.042
*t*9-18:1	3.7 ± 0.14	4.4 ± 0.44	3.9 ± 0.14	3.8 ± 0.14	4.4 ± 0.77	4.0 ± 0.22	0.593
*t*10-18:1	49.4^ab^ ± 6.19	61.3^a^ ± 5.11	47.4^ab^ ± 7.06	30.2^bc^ ± 6.98	46.1^ab^ ± 8.51	8.3^c^ ± 1.44	<0.001
*t*11-18:1	18.0^b^ ± 1.51	17.6^b^ ± 2.81	21.1^b^ ± 3.00	20.7^b^ ± 1.87	16.9^b^ ± 1.79	36.9^a^ ± 3.81	0.004
*t*12-18:1	6.2 ± 0.24	6.2 ± 0.43	6.2 ± 0.32	5.6 ± 0.65	5.5 ± 0.48	6.5 ± 0.46	0.733
*t*15-18:1	5.6 ± 0.30	5.8 ± 1.29	5.2 ± 0.52	6.2 ± 0.99	6.6 ± 1.46	6.4 ± 0.57	0.754
*t*16-18:1^3^	0.7 ± 0.03	0.6 ± 0.06	0.7 ± 0.04	0.7 ± 0.09	0.7 ± 0.06	0.8 ± 0.06	0.075
PUFA	49.5^b^ ± 1.63	51.4^ab^ ± 2.93	49.3^ab^ ± 2.15	46.2^ab^ ± 5.40	43.4^b^ ± 3.39	59.8^a^ ± 3.09	0.016
*c*9*t*13/*c*9*t*14-18:2	2.6^b^ ± 0.19	2.2^b^ ± 0.35	2.7^b^ ± 0.26	3.2^ab^ ± 0.52	2.7^b^ ± 0.38	4.9^a^ ± 0.37	<0.001
*c*9*t*15/*t*8*c*13-18:2	0.9^b^ ± 0.05	0.8^b^ ± 0.09	0.9^b^ ± 0.07	1.0^b^ ± 0.14	0.8^b^ ± 0.10	1.5^a^ ± 0.10	<0.001
*c*9*t*12-18:2	0.5 ± 0.02	0.4 ± 0.04	0.5 ± 0.03	0.5 ± 0.07	0.5 ± 0.05	0.6 ± 0.05	0.500
*t*9*c*12-18:2	0.4 ± 0.02	0.5 ± 0.10	0.4 ± 0.04	0.4 ± 0.03	0.4 ± 0.04	0.5 ± 0.04	0.055
*t*11*c*15/*t*10*c*15-18:2	1.9 ± 0.17	1.8 ± 0.21	2.2 ± 0.21	1.5 ± 0.19	1.7 ± 0.22	2.9 ± 0.49	0.102
18:2n-6	33.3 ± 1.47	37.6 ± 2.64	30.7 ± 1.94	33.9 ± 3.97	33.0 ± 2.91	31.7 ± 2.79	0.444
18:3n-6	0.2 ± 0.01	0.2 ± 0.03	0.2 ± 0.02	0.2 ± 0.04	0.3 ± 0.03	0.2 ± 0.03	0.050
18:3n-3	3.9^bc^ ± 0.32	2.3^c^ ± 0.57	4.6^b^ ± 0.42	4.2^bc^ ± 0.85	2.0^c^ ± 0.62	7.9^a^ ± 0.60	<0.001
*c*9*t*11-18:2	5.5^b^ ± 0.43	4.9^b^ ± 0.48	6.7^ab^ ± 0.87	5.4^b^ ± 0.44	4.8^b^ ± 0.46	10.3^a^ ± 1.02	<0.001
*t*10*c*12-18:2	0.5^a^ ± 0.05	0.7^a^ ± 0.09	0.5^ab^ ± 0.07	0.3^b^ ± 0.02	0.5^ab^ ± 0.09	0.4^ab^ ± 0.04	<0.001
*t*11*c*13-18:2^4^	0.3^b^ ± 0.03	0.3^b^ ± 0.03	0.4^ab^ ± 0.04	0.3^ab^ ± 0.06	0.5^ab^ ± 0.06	0.6^a^ ± 0.08	0.043
*tt*-CLA	0.3 ± 0.02	0.4 ± 0.04	0.3 ± 0.03	0.4 ± 0.05	0.4 ± 0.04	0.4 ± 0.04	0.054
LC-PUFA	2.3^b^ ± 0.10	2.0^b^ ± 0.07	2.2^b^ ± 0.18	2.1^b^ ± 0.18	2.2^b^ ± 0.18	3.1^a^ ± 0.15	<0.001
20:2n-6	0.3 ± 0.01	0.3 ± 0.02	0.3 ± 0.02	0.3 ± 0.03	0.3 ± 0.07	0.3 ± 0.02	0.524
20:3n-9^5^	0.5^b^ ± 0.05	0.3^c^ ± 0.03	0.6^ab^ ± 0.09	0.4^bc^ ± 0.02	0.4^bc^ ± 0.04	0.9^a^ ± 0.08	<0.001
20:3n-6	0.3^ab^ ± 0.01	0.3^ab^ ± 0.03	0.2^b^ ± 0.02	0.3^a^ ± 0.04	0.2^ab^ ± 0.03	0.2^ab^ ± 0.03	0.015
20:4n-6	0.6 ± 0.02	0.5 ± 0.04	0.5 ± 0.03	0.6 ± 0.07	0.6 ± 0.05	0.5 ± 0.05	0.227
20:5n-3	0.1^b^ ± 0.02	0.2^ab^ ± 0.03	0.2^ab^ ± 0.02	0.2^ab^ ± 0.05	0.2^ab^ ± 0.04	0.3^a^ ± 0.04	0.030
22:4n-6	0.1^bc^ ± 0.01	0.2^a^ ± 0.02	0.03^c^ ± 0.02	0.1^abc^ ± 0.04	0.1^ab^ ± 0.03	0.03^c^ ± 0.03	<0.001
22:5n-3	0.5^b^ ± 0.04	0.3^c^ ± 0.03	0.5^bc^ ± 0.07	0.5^bc^ ± 0.05	0.4^bc^ ± 0.07	1.0^a^ ± 0.06	<0.001
Other	0.4^b^ ± 0.03	0.5^b^ ± 0.06	0.5^b^ ± 0.04	0.4^b^ ± 0.09	0.5^b^ ± 0.07	0.8^a^ ± 0.06	<0.001
Cyclo-17^6^	0.4^b^ ± 0.03	0.5^b^ ± 0.06	0.5^b^ ± 0.04	0.4^b^ ± 0.09	0.5^b^ ± 0.07	0.8^a^ ± 0.06	<0.001
Sums or ratio							
*t*10-shift	4.7^a^ ± 0.84	4.9^a^ ± 0.71	3.9^ab^ ± 0.80	1.7^bc^ ± 0.49	3.3^ab^ ± 0.82	0.2^c^ ± 0.05	<0.001
BI^7^	109^ab^ ± 5.8	119^a^ ± 4.7	112^ab^ ± 5.7	93.2^b^ ± 4.6	103^ab^ ± 9.8	98.2^ab^ ± 7.6	0.021

Letters in the same row with different superscripts are statistically different (*P* < 0.05).

1 Coelutes with a-17:0.

2 Coelutes with *t*13/*t*14/*c*10-18:1 as minor isomers.

3 Coelutes with *c*14-18:1 as minor isomer.

4 Coelutes with 21:0.

5 Coelutes with *c*9*t*11*c*15-18:3.

6 Methyl-11-cyclohexylundecanoate.

7 Sum of all C18 FA excluding 18:0, *c*9-18:1, *c*11-18:1, 18:2n-6, 18:3n-6 and 18:3n-3.

Total BCFA ranged from 1.07 to 1.3 % and tended (*P* = 0.076) to be higher in X-Suffolk, particularly due to higher iso17:0 (*P* = 0.004). Total MUFA represented the largest fraction of unsaturated FA (39.1–42.2 %) and were not significantly affected by the genetic group (*P* = 0.161). Although total *cis*-MUFA did not differ between groups, total *trans*-MUFA did, with the lowest values observed in X-Suffolk and X-Merino (7.16 % and 7.5 % of total FA, respectively). Also, marked differences were observed in several *cis* and *trans*-18:1 isomers, including *t*10-18:1, *t*11-18:1, *c*9-18:1 and *c*11-18:1 (*P* < 0.01). Interestingly, the X-Suffolk showed the lowest *t*10-18:1 proportion (0.8 ± 0.14 % of total FA) and the highest *t*11-18:1 proportion (3.7 ± 0.38 % of total FA) in adipose tissue. As a result, X-Suffolk was the only genetic group with a *t*10-shift ratio below 1 (0.2 ± 0.05), but it did not differ from the X-Merino lambs, which had a *t*10-shift ratio of 1.7 ± 0.49.

The PUFA accounted for 4.3–6.0 % of total FA and differed among groups (*P* = 0.016), with the highest values found in X-Suffolk lambs (5.98 % of total FA). The major PUFA was the 18:2n-6, however, it did not differ among genetic groups (*P* = 0.444). In contrast, individual BH intermediates, such as *c*9*t*11-18:2, differed significantly, with X-Suffolk showing the highest value of 1.03 % of total FA. The X-Suffolk lambs also showed the highest proportions of 18:3n-3, LC-PUFA, 20:5n-3 and 22:5n-3, with 0.79 %, 0.31 %, 0.03 % and 0.1 % of total FA, respectively.

**Fig. 5:**
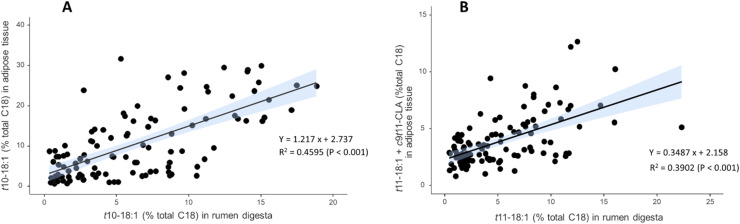
Relationships between (A) *trans*-10 18:1 in rumen digesta and adipose tissue, i.e. kidney knob channel fat (% of total C18 fatty acids) and (B) *trans*-11 18:1 in rumen digesta and *t*11-18:1 + *c*9*t*11-CLA in adipose tissue, kidney knob channel fat (% of total C18 fatty acids). Each point represents an individual sample. Solid lines indicate linear regression fits with 95 % confidence intervals. Regression equations, coefficients of determination (R²), and significance levels are shown within each panel.

The relationship between the FA composition of rumen digesta and KKCF was also explored, specifically on the BH intermediates *t*10-18:1 and *t*11-18:1 (Fig. 5). For *t*11-18:1, the relationship was assessed using the concentration of *t*11-18:1 in rumen digesta and the sum of *t*11-18:1 and *c*9*t*11-18:2 in KKCF, since, after absorption, *t*11-18:1 can be converted to *c*9*t*11-18:2 by the action of the Δ9-desaturase enzyme. The figure shows that the contents of *t*10-18:1 and t11-18:1 in rumen digesta are positively associated with the proportions of *t*10-18:1 and *t*11-18:1 + *c*9*t*11-18:2 in KKCF, respectively. The relationship was stronger for *t*10-18:1 (R² = 0.46; *P* < 0.001) than for *t*11-18:1 (R² = 0.39; *P* < 0.001).

## 4 Discussion

Although the occurrence of the *t*10-shift is well known in lambs fed high-starch, low-forage diets (Alves et al., 2021; Bessa et al., 2015), no study has systematically estimated its prevalence in the rumen of large-scale field populations. Indeed, most studies on the ruminal *t*10-shift use controlled experiments with a limited number of animals or rely on surveys of retail meat or fat samples, rather than proper surveys of field populations. Nonetheless, these studies reveal substantial individual variability in the expression of the *t*10-shift even among animals fed identical diets. However, most of the studies used adipose tissue (Aldai et al., 2009; Bessa et al., 2015; Bravo-Lamas et al., 2016; Santos-Silva et al., 2020, 2023) or milk (Dewanckele et al., 2020b; Jing et al., 2018; Toral et al., 2020) levels of *t*10-18:1, *t*10*c*12-CLA and *t*10-18:1/*t*11-18:1 ratio as proxies of rumen *t*10-shift, rather than directly analysing rumen digesta samples. Thus, this work was initially designed to evaluate the prevalence of *t*10-shift in the rumen, in a large population of lambs (*n* = 611) finished under commercial standard conditions, and to identify animals that may be resistant to this alteration. The animals were finished and managed under the same conditions, i.e., on the same finishing company, and all were fed *ad libitum* a high-concentrate diet to ensure identical conditions. Before the finishing phase, animals were sourced from multiple ewe-lamb farms in southern Portugal to obtain a large and representative sample of the types of animals available for commercial finishing. Although this type of survey is very useful for gathering information on a large population maintained under current production practices, it fails to control several production variables, such as feed intake, growth rate, and rumen pH, measured under standardised conditions, which would be of great interpretative value. Furthermore, the sampled animals reflect the availability of animals for finishing and include both purebred and crossbred lambs, with unequal representation among genetic groups. These limitations should be taken into account, and only cautious interpretations of the effects of the genetic group effects can be made.

Our results show that the prevalence of the *t*10-shift in the rumen, defined as the ratio *t*10-/*t*11-18:1 > 1, is 90 %. In fact, >80 % of animals exhibit a *t*10-/*t*11-18:1 > 2, thus expressing a very evident *t*10-shift. The amplitude of the ratio was also very large, as presented in Fig. 2. These results are consistent with several previous controlled experiments in which animals were fed high-concentrate diets (Bessa et al., 2015). Also, in a survey of commercially available lamb meat in Northern Spain (with 24 samples collected in spring and 24 in winter), two groups based on their *t*10-/*t*11-18:1 ratio in subcutaneous fat were also identified, with 21 samples presenting a ratio ≤1 and 27 a ratio > 1 (Bravo-Lamas et al., 2016). A survey of the FA composition of Canadian beef also identified that the most abundant BH intermediate in backfat and *Longissimus lumborum* muscle was the *t*10-18:1 (Aldai et al., 2009). In those studies, the *t*10-shift was estimated from the proportions of *t*10-18:1 and *t*11-18:1 in ruminant tissues, assuming that these proportions reflect those that leave the rumen and are deposited in tissues. Indeed, in a previous study (Alves et al., 2017), we showed significant correlations between the *t*10-shift in the rumen digesta and the *t*10-shift in both the abomasal digesta (r^2^ = 0.891, *P* < 0.001) and plasma (r^2^ = 0.754, *P* < 0.001). In the present work, we also observed significant correlations between *t*10-18:1 and *t*11-18:1 in rumen digesta, and with the *t*10-18:1 and *t*11-18:1 + *c*9*t*11-18:2 in adipose tissue, suggesting that the proportions of these FA in tissues can be used to estimate the occurrence of the *t*10-shift in the rumen. However, it is important to note that rumen digesta FA composition only captures the state at the moment of slaughter, whereas the tissues, including KKCF, the FA composition reflects the initial tissue concentration and the cumulative effect of deposition over the entire finishing period. Indeed, in tissues, the *t*11-18:1 can be converted into *c*9*t*11-18:2 by the enzyme stearoyl-CoA desaturase (SCD), which can overestimate the *t*10-shift computed as *t*10-18:1/*t*11-18:1 ratio in tissues. This explains why the relationships between rumen digesta and KKFC were computed using the proportions of *t*10-18:1 and *t*11-18:1, plus *c*9*t*11-18:2, rather than the ratio *t*10-18:1/*t*11-18:1.

Nevertheless, it is notable that despite the strong dietary stimulus favouring the establishment of the *t*10-shift, 65 animals are putatively resistant as they maintain a *t*10-18:1/*t*11-18:1 ratio below 1. The X-Suffolk group presented the highest proportion of *t*10-shift resistant lambs (37 %, i.e. 15 out of 41), whereas the Merino and X-ILFrance were highly susceptible, with only 7 % of total Merino and 5 % of total X-ILFrance lambs not shifting. Differences among breeds in the expression of *t*10-shift in the rumen digesta have not been clearly demonstrated, but the study of Costa et al. (2013) suggested that the Barrosã bovine breed showed less variability and a lower *t*10-/*t*11-18:1 ratio in meat than the Alentejana breed (Bessa et al., 2015; Costa et al., 2013b). Our results also suggest that susceptibility to the *t*10-shift might be influenced by genetic background.

Nevertheless, conclusions about breed or genotype effects should be drawn with caution, as these effects may be confounded by other factors, such as sampling season, variation in live weight, slaughter age, dietary intake, or feed management before finishing, including pasture availability, pasture quality, and concentrate supplementation. Information collected on lamb feeding management before finishing indicated that some lambs were already consuming concentrate, whereas others remained with their mothers on pasture. Correlations between pre-finishing diet and *t*10-shift markers were explored, but no significant associations were found, probably due to the diversity of management systems and the difficulty of grouping animals. Nevertheless, our results suggest that the feeding system before finishing did not affect the occurrence of the *t*10-shift. For example, in sampling 11, according to our records, all lambs grazed on pasture with their mothers, even so, the variability in the *t*10-shift in rumen digesta was very large (Fig. 2). Conversely, in sampling 8, which showed the lowest *t*10-shift values, most animals had already started consuming concentrate. Data on live weight variation, slaughter age, and dietary intake during the finishing period were not available. However, lambs were typically weaned at 45–60 days of age, and the finishing period lasted 45–50 days before slaughter. Regarding the cold carcass weight, no differences were detected among genetic groups, and no relationships were found between cold carcass weight and the *t*10-shift ratio.

Thus, while conclusions about the effect of the genetic group should be interpreted with caution, X-Suffolk lambs showed not only the highest frequency of low *t*10-shift values (<1) but also the highest rumen pH values. This is consistent with studies reporting that the occurrence of *t*10-shift in the rumen is often associated with low rumen pH in several *in vitro* (Troegeler-Meynadier et al., 2014), in vivo (Colman et al., 2012), and in continuous culture studies (Fuentes et al., 2009). In our experiment, the Spearman correlations also showed significant negative associations of pH with *t*10-18:1 and *t*10-shift and positive associations with *t*11-18:1. *In vitro* studies from our group with rumen inoculum and pure *t*10-18:1 and *t*11-18:1 at high and low pH, showed that at low pH, BH of *t*10-18:1 proceeds more slowly than *t*11-18:1, which helps to explain its accumulation under acidic conditions (Fernandes et al., 2024). These results are consistent with the negative correlation of pH with the total BH intermediates, and the positive correlation of pH with 18:0, as presented in Fig. 3. This means that at lower pH, BH intermediates accumulate, including the *t*10-18:1, while the 18:0, the end-product of BH, decreases. In fact, significant (*P* < 0.001) regressions could be established between *t*10-18:1 and 18:0 (r^2^=0.58, 18:0 = 56.1–1.03*(*t*10-18:1)), as well as *t*10-18:1 and BH intermediates (r^2^=0.81, BH intermediates = 6.24+1.117*(*t*10-18:1)), showing negative and positive slopes, respectively (Fig. 4). However, considering that the *t*10-18:1 is the major BH intermediate, these results might be biased. Regarding the correlation between *t*11-18:1 and 18:0, although it was significant, the R² was only 0.1287. The accumulation of BH intermediates results from decreased ruminal BH completeness, often due to the inhibition of the last step of rumen BH (reduction of 18:0 formation), which might be associated with inhibition of 18:0-producing bacteria or the relative increase in number and activity of more acid-tolerant *t*10-producing bacteria (Dewanckele et al., 2020a, 2018; Lv et al., 2016).

Interestingly, several significant correlations between *t*10-18:1 and other BH intermediates were also observed. The positive correlation between *t*10-18:1 and the *t*6- to *t*9-18:1 isomers, along with the negative correlation with *t*15-, *t*16-, and *c*16-18:1 isomers, might indicate that the BH pathways favouring *t*10 production also promote the formation of intermediates with *trans* double bonds near the carbonyl group, rather than those with double bonds towards the end of the carbon chain. In the study by Bravo-Lamas et al. (2016), samples of lamb subcutaneous fat were classified according to the *t*10-/*t*11-18:1 ratio (>1 or <1), and those with a ratio >1 also showed higher contents of *t*6/*t*7/*t*8-18:1 and *t*9-18:1, whereas samples with a ratio <1 showed higher contents of *t*16- and *t*15-18:1. Thus, our findings, along with the positive correlations of *t*6- to *t*10- isomers with oleic acid (*c*9-18:1) and the lack of correlations of *t*10-18:1 with both 18:2n-6 and 18:3n-3, might support the hypothesis that these *trans*-18:1 isomers are largely formed from the ruminal BH of oleic acid. Indeed, AbuGhazaleh et al. (2005) showed, in continuous-flow ruminal fermenters with ^13^C-oleic acid, that under conditions of low pH (5.6) and low dilution rate, the conversion of oleic acid to *trans*-18:1 is limited to double-bond positions from C6 to C10, whereas under higher pH (6.5) and higher dilution rate, ^13^C derived from oleic acid appears in *trans*-18:1 isomers from C6 to C16. Again, the negative correlations of pH with *t*6- to *t*10-18:1 isomers and the positive correlations with *t*15- and *t*16-18:1 isomers are consistent with those findings.

Jenkins et al. (2006) also demonstrated through *in vitro* cultures that ruminal microorganisms can transform oleic acid into 10-ketostearic acid (10-oxo-18:0), and that its accumulation in the rumen contents is directly related to the amount of oleic acid provided and its metabolization. Moreover, McKain et al. (2010) showed that the ruminal bacterium *Propionibacterium acnes* can convert not only oleic acid, but also *t*10-18:1, into 10-oxo-18:0. Thus, the positive associations of 10-oxo-18:0 with both *t*10-18:1 and *c*9-18:1 are consistent with those studies. In addition, the negative correlations of 10-oxo-18:0 with 18:0, *t*15- and *t*16-18:1 isomers, and pH, together with its positive correlations with *t*6/*t*7/t8-18:1 and the *t*10-shift, suggest that the conditions and microbiota associated with the *t*10-shift also favour the formation of 10-oxo-18:0 in the rumen. However, it is important to note that, under our chromatographic conditions, *c*9-18:1 could not be resolved from the *t*13-/*t*14-18:1 isomer pair, and therefore, the correlations involving *c*9-18:1 may have been affected by the presence of these isomers.

The changes in the BH pathways in the rumen are often associated with alterations in fermentation patterns and rumen microbiota. Specifically, high-starch diets and low rumen pH are associated with a decrease in the acetate-to-propionate ratio, indicating a shift toward propionate-type fermentation (Wang et al., 2020). In accordance with this, we observed positive associations between pH and 2:0/3:0 ratio (Supplementary Figure S4). The associations of pH, *t*10-shift and *t*10-18:1 with several DMA and odd and branched-chain fatty acids (OBCFA), from microbial origin, also suggest changes in the rumen community structure or activity. In fact, rumen bacterial membranes are enriched in OBCFA, synthesised *de novo* from propionate and branched‑chain VFA derived from branched‑chain amino acids fermentation (Fievez et al., 2012). DMA derived from plasmalogen lipids in anaerobic bacterial lipids, which also contain odd- and branched-chain moieties, was suggested to also serve as indirect markers of the rumen microbial community (Alves et al., 2013). Indeed, some bacterial genera were reported to be strongly and differentially correlated with multiple DMA species and with odd‑ and branched‑chain FA, confirming that these lipid classes are sensitive markers of bacterial composition (Conte et al., 2022; Cremonesi et al., 2018).

Genotype-specific differences were found in VFA composition in rumen content, as well as in DMA and OBCFA composition, indicating variations in the rumen microbiota. Since the diet during the finishing period was constant across genetic groups, these findings imply that diet during the pre-finishing phase or host genetics may influence microbiome composition and function, thereby affecting VFA and microbial marker levels, i.e., OBCFA and DMA. Previous studies have shown that forage-based diets increase iso-DMA, iso-BCFA (Alves et al., 2013), and total OBCFA concentrations in rumen digesta, whereas diets richer in concentrate and starch increase linear odd-chain FA and decrease iso-BCFA (Vlaeminck et al., 2006), indicating shifts in microbial populations, with BCFA and branched-chain DMA playing important roles in bacterial function and growth (Mitchell et al., 2023). In our study, Romane lambs showed the lowest DMA content in rumen digesta, while Merino×Lacaune lambs showed the highest. It is tempting to believe that these differences could arise from the feeding management before the finishing phase because, as Romane lambs had already been receiving concentrate before the finishing period, while Merino×Lacaune lambs stayed with their mothers and had access to pasture, although the finishing period will certainly reduce that effect. Similarly, X-Suffolk lambs, which had received concentrate before the finishing period, showed the lowest BCFA content, including the lowest iso-BCFA levels, compared with Merino lambs, some of which remained with their mothers on pasture prior to finishing.

Thus, these results are consistent with the literature that suggests that diet and management largely shape rumen metabolite profiles. Nevertheless, host genotype can significantly modulate these aspects, as even under the same feeding management and diets, genetic influences on specific taxa, functions, and fermentation pathways have been consistently reported (Abbas et al., 2020; Hess et al., 2023; Li et al., 2019). The largest differences in rumen FA and C18 FA content among genetic groups support the link between the rumen environment and genotype-dependent lipid metabolism.

When examining differences in FA composition in KKCF among genetic groups, it is evident that variation in microbial-origin FA is less pronounced than in rumen digesta. For example, among the BCFA, only i-17:0 differed between genetic groups, and no differences were observed in total linear SFA. In contrast, greater variation was found in PUFA, including C18 FA and LC-PUFA, with X-Suffolk showing the highest proportion of LC-PUFA, particularly n-3 PUFA. Nevertheless, compared with rumen digesta, adipose tissue reflects the cumulative deposition of FA over time. Therefore, although the finishing diets were similar among genetic groups and X-Suffolk lambs had already been receiving concentrate prior to finishing, genetic background may also have influenced FA deposition in adipose tissue, particularly n-3 PUFA elongation and desaturation. Previous studies in cattle have shown that the FA composition of subcutaneous adipose tissue and *longissimus lumborum* muscle depends largely on genetic background (Costa et al., 2013a). Another study has also shown that the double-muscled genotype significantly alters the elongation and desaturation products of n-3 PUFA in intramuscular fat, even under a single, fixed diet (Aldai et al., 2008).

## 5 Conclusions

The main conclusions highlight the crucial role of diet and intensive systems in promoting the *trans*-10 shift in finishing lambs, in which *trans*-10 18:1 predominates over *trans*-11 18:1. This metabolic alteration negatively affects the nutritional quality of ruminants' edible fats and, consequently, human health by potentially increasing the risk of cardiovascular disease. Furthermore, the observation that about 10 % of animals were resistant to the *trans*-10 shift suggests that selective breeding for this trait could enhance the nutritional quality of intensively finished lambs. Notably, the crossed Suffolk lambs showed the highest frequency of *t*10-shift-resistant animals, justifying further research using a larger population of purebred Suffolk animals. Despite substantial variability within and among genetic groups, *trans*-10 shift-resistant animals occur across all genetic groups, and this variability warrants further investigation. This phenotyping work is part of a larger project in which genome-wide association studies will be applied to identify a genetic marker for the *t*10-shift trait.

## Ethical statement

All conditions and procedures conducted with the animals are standard animal husbandry practices carried out in accordance with Portuguese (Decreto-Lei 113/2013) and EU (Directive 2010/63/EU) legislation on animal experimentation and welfare.

## Funding

This work was funded by 10.13039/501100001871Fundação para a Ciência e a Tecnologia (FCT) through the research projects PTDC/CAL-ZOO/4515/2021 (Gene2Rumen), UID/00276/2025 (CIISA), UIDB/05183/2025 (MED), LA/P/0059/2020 (AL4Animals), and LA/P/0121/2020 (CHANGE) and the PhD research grant to CM (UI/BD/152816/2022).

## Data availability

Data in the current study are available upon reasonable request.

## CRediT authorship contribution statement

**Chica Manuel:** Writing – review & editing, Writing – original draft, Methodology, Investigation, Formal analysis. **Cristina Xavier:** Methodology, Investigation, Formal analysis. **Saeedeh Moradi:** Methodology, Investigation, Formal analysis. **Diana M. Soares:** Methodology, Investigation, Formal analysis. **Aristides Manuel:** Methodology, Investigation, Formal analysis. **Liliana Cachucho:** Methodology, Investigation, Formal analysis. **Letícia Fialho:** Methodology, Investigation, Formal analysis. **David Soldado:** Methodology, Investigation, Formal analysis. **Andreia Silva:** Methodology, Investigation, Formal analysis. **Patrícia Lage:** Methodology, Investigation, Formal analysis. **Olinda Guerreiro:** Writing – review & editing, Methodology, Investigation, Formal analysis. **Cármen Garrine Bule:** Methodology, Investigation, Formal analysis. **Eliana Jerónimo:** Writing – review & editing, Project administration, Methodology, Investigation, Funding acquisition, Formal analysis, Conceptualization. **Rui J.B. Bessa:** Writing – review & editing, Project administration, Methodology, Investigation, Funding acquisition, Formal analysis, Conceptualization. **Susana P. Alves:** Writing – review & editing, Writing – original draft, Validation, Supervision, Project administration, Methodology, Investigation, Funding acquisition, Formal analysis, Data curation, Conceptualization.

## Declaration of competing interest

The authors declare the following financial interests/personal relationships which may be considered as potential competing interests:

Given her role as Associate Editor of the Veterinary and Animal Science journal, Susana P. Alves had no involvement in the peer review of this article and had no access to information regarding its peer review. Full responsibility for the editorial process for this article was delegated to another journal editor. If there are other authors, they declare that they have no known competing financial interests or personal relationships that could have appeared to influence the work reported in this paper.

## Appendix Supplementary materials

Image, application 1
