# Supplementary material for: Phenotyping fatty acids and the trans-10 shift in the rumen content and adipose tissue of commercial finishing lambs
Source: Vet Anim Sci. 2026 Apr 15;32:100662. doi: 10.1016/j.vas.2026.100662 (PMC13123586; doi:10.1016/j.vas.2026.100662)
Supplement: Supplementary file 1 [file mmc1.pdf]

Supplementary Tables and Figures

Phenotyping fatty acids and the *trans*-10 shift in the rumen content and adipose tissue of commercial finishing lambs

Table S1 - Description of the study population, sampling design, and sampling dates. A total of 630 animals were included in the study (19 animals were excluded after quality control). Animals originated from 24 farms, representing seven genetic groups and seven counties. Samples were collected at 11 sampling dates between June 2022 and July 2024.

Description	Number
Animals	630 (19 were excluded) = 611
Ewe-Lamb Farms	24
Genetic group	7
Merino	250
Romane	43
X-ILFrance[1]	134
Merino×Lacaune	14
X-Merino[1]	49
X-Romane[1]	80
X-Suffolk[1]	41
County	7
Sampling	11
1 - June 2022	40
2 - July 2022	48
3 - September 2022	60
4 - November 2022	60
5 - January 2023	59
6 - February 2023	59
7 - May 2023	59
8 - July 2023	60
9 - September 2023	56
10 - February 2024	60
11 - July 2024	50

[1]Ewes that do not have a clear breed affiliation were crossed (X) with purebred rams.

Table S2 – Feed management before finishing period according to Sampling data, genetic group, county and farm.

Sampling	Genetic group	County	Farm	Description of feed management before the finishing period	n
1	X-ILFrance	Odemira	Farm 1	Lambs with their mothers in the field. Ewes supplemented with concentrate. Lambs received concentrate	3
	X-Merino	Ourique	Farm 2	Weaned lambs eating concentrate	15
	X-Romane	Ourique	Farm 3	Lambs with their mothers in the field. Supplementation of mothers with concentrate (clubs). Lambs were already receiving concentrate	14
	X-Suffolk	Ourique	Farm 4	Lambs with their mothers in the field. Ewes supplemented with concentrate. Lambs received concentrate	8
2	X-Merino	Ourique	Farm 2	Weaned lambs eating concentrate	18
	X-Merino	Ourique	Farm 4	Lambs with their mothers in the field. Ewes supplemented with concentrate. Lambs received concentrate	1
	X-Romane	Ourique	Farm 3	Lambs with their mothers in the field. Supplementation of mothers with concentrate (clubs). Lambs were already receiving concentrate	19
	X-Suffolk	Ourique	Farm 2	Weaned lambs eating concentrate	4
	X-Suffolk	Ourique	Farm 4	Lambs with their mothers in the field. Ewes supplemented with concentrate. Lambs received concentrate	8
3	Merino	Ourique	Farm 5	Lambs with their mothers in the field. Ewes supplemented with concentrate. Lambs are separated every day (4-5 hours/day) to receive concentrate	16
	Merino	Ourique	Farm 6	Lambs with the sheep. Ewes supplemented with concentrate. Lambs received concentrate	14
	X-ILFrance	Almodovar	Farm 7	Lambs with their mothers in the field (without pasture). Mothers fed concentrate and forage. Lambs had already been provided with concentrate	12
	X-Romane	Ourique	Farm 8	Lambs with their mothers in the field (without pasture). Mothers fed concentrate and forage. Lambs had already been provided with concentrate	18
4	Merino	Ourique	Farm 9	Lambs with their mothers in the field. Ewes supplemented with concentrate. Lambs are separated every day (4-5 hours/day) to receive concentrate	12
	Merino	Ourique	Farm 10	Lambs with the sheep in the field. No supplementation of the ewes. Lambs were already receiving concentrate	8
	X-ILFrance	Mértola	Farm 11	Lambs with their mothers in the field. Ewes supplemented with concentrate. Lambs received concentrate	40
5	X-ILFrance	Odemira	Farm 1	Lambs with their mothers in the field. Pasture available	30
	M×Lacaune	Serpa	Farm 12	Lambs with their mothers. Pasture available	14
	X-Merino	Mértola	Farm 13	Lambs with sheep in the field. Lambs were already receiving concentrate	16
6	Merino	Beja	Farm 14	Lambs with the sheep in the field. No supplementation of the ewes. Lambs still did not receive concentrate	22
	X-ILFrance	Serpa	Farm 15	Lambs with their mothers in the field. Pasture available	17
	X-Suffolk	Ourique	Farm 16	Lambs with their mothers in the field. No supplementation for mothers. Lambs were already receiving concentrate	21
7	Romane	Odemira	Farm 17	Ewes trapped from farrowing to weaning, fed concentrate and hay. Lambs were already receiving concentrate	15
	X-ILFrance	Odemira	Farm 18	Lambs with their mothers in the field. No supplementation for mothers. Lambs did not receive concentrate	34
	X-Romane	Castro Verde	Farm 19	Weaned lambs eating concentrate	11
8	Merino	Almodovar	Farm 20	Lambs with their mothers in the field. They walked on stubble (without supplementation of sheep and lambs)	22
	Romane	Castro Verde	Farm 21	Weaned lambs eating concentrate	18
	X-Romane	Ourique	Farm 22	Lambs with sheep in the field, with access to grain cereal fodder. Lambs received concentrate	20
9	Merino	Ourique	Farm 5	Lambs with their mothers in the field. Ewes supplemented with concentrate. Lambs are separated every day (4-5 hours/day) to receive concentrate	50
	Merino	Ourique	Farm 23	Lambs with sheep in the field (no pasture available). Ewes supplemented with concentrate. Lambs are given concentrate	30
	Romane	Odemira	Farm 17	Ewes trapped from farrowing to weaning, fed concentrate and hay. Lambs were already receiving concentrate	10
10	Merino	Ourique	Farm 5	Lambs with their mothers in the field. Ewes supplemented with concentrate. Lambs are separated every day (4-5 hours/day) to receive concentrate	43
	Merino	Ourique	Farm 2	Lambs with their mothers. Only pasture	17
11	Merino	Almodovar	Farm 20	Lambs with their mothers. Only pasture	20
	Merino	Beja	Farm 14	Lambs with their mothers. Only pasture	17
	Merino	Mértola	Farm 24	Lambs with their mothers. Only pasture	23

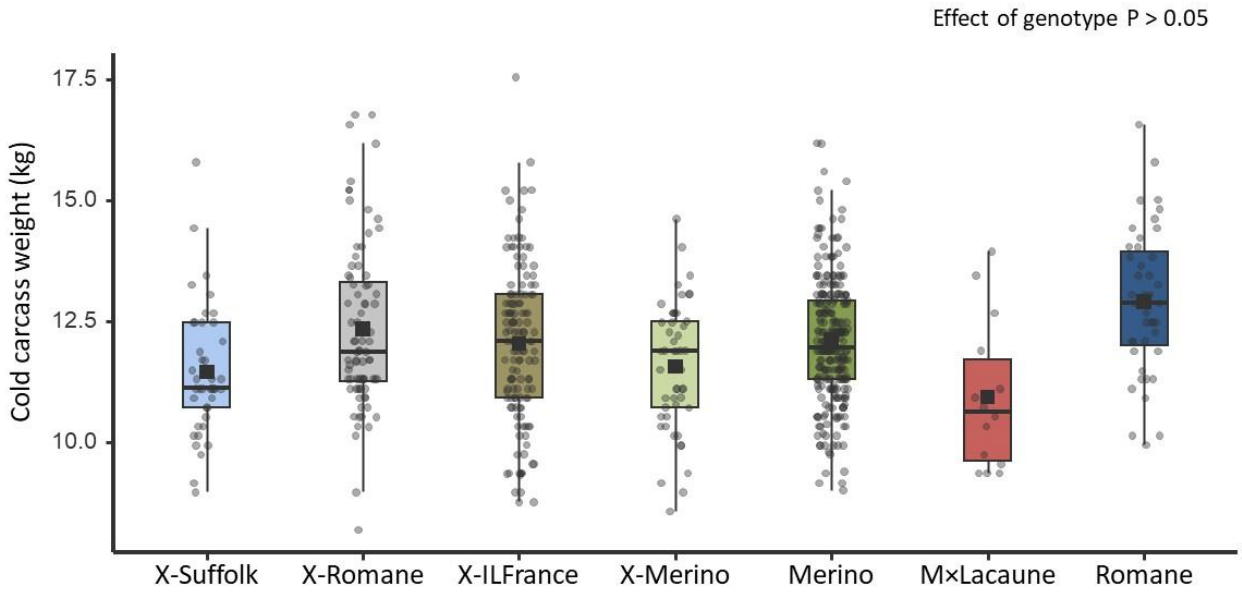

Figure S1 - Distribution of cold carcass weight (kg) according to genetic group. Boxplots represent the median (horizontal line), interquartile range (box), mean (square dot) and minimum–maximum values. Individual observations are shown as jittered points.

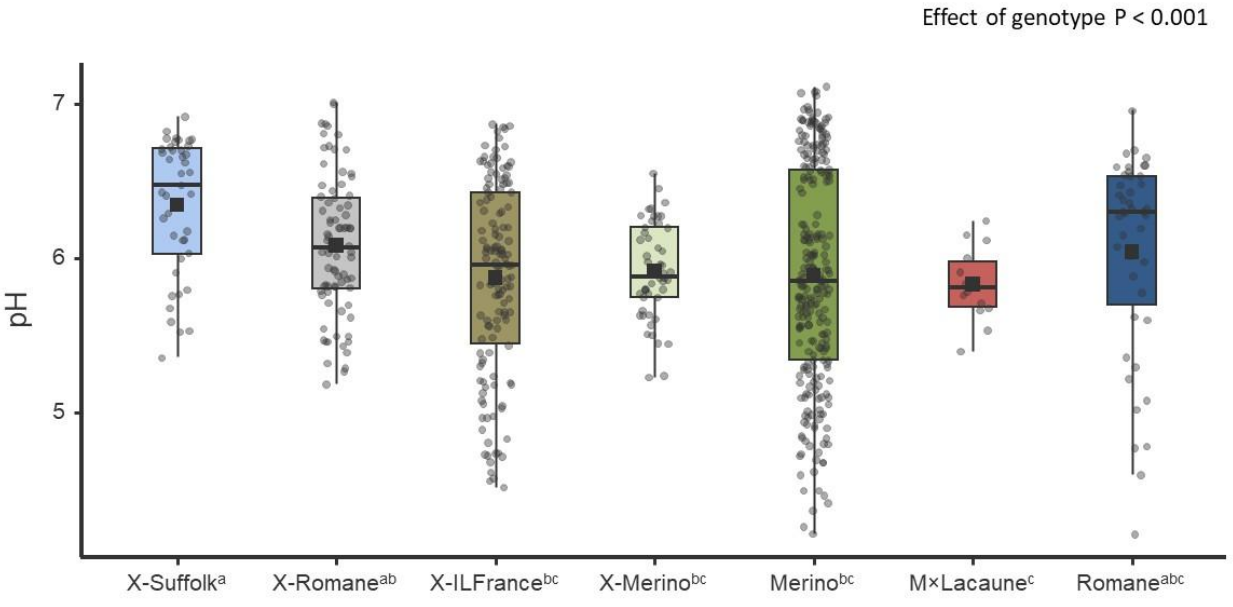

Figure S2 - Rumen pH distribution according to genetic group. Boxplots represent the median (horizontal line), interquartile range (box), mean (square dot) and minimum–maximum values. Individual observations are shown as jittered points. Different superscript letters indicate significant differences among genotypes ($P < 0.05$).

Table S3 - Descriptive statistics of *trans*-10 18:1 (*t*10-18:1) and *trans*-11 18:1 (*t*11-18:1) contents (% of total C18 fatty acids) in rumen content according to genetic group. Data presented are the number of observations (N), mean, standard error of the mean (SEM), variance, amplitude (range), minimum, and maximum values.

Genetic group	N	Average	SEM	Variance	Amplitude	Minimum	Maximum
***t*10-18:1**							
X-Suffolk	41	12.89	1.267	65.802	29.28	0.620	29.90
X-Romane	80	12.37	0.864	59.784	36.90	0.990	37.89
X-ILFrance	134	15.06	0.741	73.543	34.63	0.910	35.54
X-Merino	49	14.10	1.076	56.686	37.28	0.580	37.86
Merino	250	13.79	0.470	55.121	34.58	1.040	35.62
M×Lacaune	14	15.78	1.961	53.824	25.18	8.880	34.06
Romane	43	10.25	0.945	38.413	25.45	1.050	26.50
***t*11-18:1**							
X-Suffolk	41	7.43	0.976	39.063	23.38	0.400	23.78
X-Romane	80	3.35	0.288	6.618	12.27	0.330	12.60
X-ILFrance	134	2.99	0.262	9.210	15.75	0.470	16.22
X-Merino	49	2.32	0.289	4.095	8.23	0.340	8.57
Merino	250	3.07	0.174	7.543	15.58	0.420	16.00
M×Lacaune	14	1.70	0.150	0.316	2.19	0.680	2.87
Romane	43	3.94	0.473	9.613	13.98	0.700	14.68

Table S4 - Spearman’s rank correlation coefficients (ρ) among C18 fatty acids in the rumen, sum of biohydrogenation intermediates (BI), and rumen pH (*, **, *** indicate significance at P < 0.05, P < 0.01, and P < 0.001, respectively).

	Spearman's Rho	18:0	t6/7/8-18:1	t9-18:1	t10-18:1	t11-18:1	t12-18:1	t15-18:1	t16-/c14-18:1	c9-18:1	c11-18:1	c12-18:1	c13-18:1	c15-18:1	c16-18:1	18:2n-6	t11c15-18:2	c9t11-CLA	t10c12-CLA	18:3n-3	c9t11c15-18:3	10-O-18:0	15-O-18:0	t10-shift	BI	pH
18:0	Spearman's Rho	—																								
t6/7/8-18:1	Spearman's Rho	-0.663***	—																							
t9-18:1	Spearman's Rho	-0.419***	0.736***	—																						
t10-18:1	Spearman's Rho	-0.822***	0.649***	0.341***	—																					
t11-18:1	Spearman's Rho	-0.346***	0.049	0.064	-0.078	—																				
t12-18:1	Spearman's Rho	-0.181***	0.243***	0.312***	-0.054	0.245***	—																			
t15-18:1	Spearman's Rho	0.195***	-0.092*	0.023	-0.374***	0.110**	0.627***	—																		
t16-/c14-18:1	Spearman's Rho	0.420***	-0.367***	-0.173***	-0.656***	0.230***	0.322***	0.601***	—																	
c9-18:1	Spearman's Rho	-0.647***	0.328***	0.254***	0.360***	0.279***	0.400***	0.219***	0.023	—																
c11-18:1	Spearman's Rho	-0.548***	0.549***	0.334***	0.598***	-0.098*	0.110**	-0.252***	-0.488***	0.257***	—															
c12-18:1	Spearman's Rho	-0.202***	0.132**	0.106**	-0.044	0.276***	0.372***	0.317***	0.206***	0.295***	0.067	—														
c13-18:1	Spearman's Rho	-0.164***	0.280***	0.216***	0.166***	-0.099*	0.174***	0.049	-0.145***	0.148***	0.226***	0.085*	—													
c15-18:1	Spearman's Rho	-0.209***	0.183***	0.000	0.199***	0.044	0.102*	0.176***	-0.110**	0.243***	0.055	0.252***	0.084*	—												
c16-18:1	Spearman's Rho	0.132**	-0.01	0.052	-0.178***	-0.012	0.210***	0.248***	0.267***	-0.014	-0.023	0.184***	0.191***	-0.063	—											
18:2n-6	Spearman's Rho	-0.308***	-0.038	-0.031	-0.073	0.436***	0.257***	0.282***	0.340***	0.612***	-0.017	0.470***	-0.048	0.124**	0.107**	—										
t11c15-18:2	Spearman's Rho	-0.661***	0.397***	0.168***	0.553***	0.307***	0.151***	-0.018	-0.193***	0.466***	0.271***	0.308***	0.029	0.388***	-0.025	0.338***	—									
c9t11-CLA	Spearman's Rho	-0.061	0.202***	0.066	0.223***	-0.264***	-0.034	-0.233***	-0.297***	-0.170***	0.440***	-0.06	0.094*	-0.011	0.082*	-0.282***	-0.064	—								
t10c12-CLA	Spearman's Rho	-0.183***	0.244***	0.156***	0.142***	0.012	0.195***	0.03	-0.004	0.123**	0.267***	0.246***	0.199***	0.042	0.310***	0.174***	0.169***	0.306***	—							
18:3n-3	Spearman's Rho	-0.200***	-0.102*	-0.145***	-0.072	0.329***	0.241***	0.357***	0.321***	0.492***	-0.135***	0.373***	-0.104*	0.346***	0.048	0.693***	0.346***	-0.288***	0.083*	—						
c9t11c15-18:3	Spearman's Rho	-0.192***	0.143***	0.049	0.238***	-0.102*	0.003	-0.184***	-0.209***	0.053	0.279***	0.126**	0.087*	0.018	0.067	0.007	0.122**	0.341***	0.344***	-0.099*	—					
10-O-18:0	Spearman's Rho	-0.695***	0.536***	0.290***	0.724***	0.079	-0.06	-0.418***	-0.574***	0.300***	0.437***	-0.124**	0.158***	0.139***	-0.258***	-0.145***	0.378***	0.147***	0.025	-0.128**	0.153***	—				
13-O-18:0	Spearman's Rho	-0.155***	0.107**	0.023	0.044	0.194***	0.093*	0.150***	0.099*	0.174***	0.045	0.332***	0.063	0.144***	0.152***	0.281***	0.285***	-0.181***	0.244***	0.226***	0.043	-0.013	—			
t10-shift	Spearman's Rho	-0.145***	0.290***	0.140***	0.555***	-0.845***	-0.208***	-0.286***	-0.516***	-0.036	0.387***	-0.233***	0.160***	0.01	-0.079	-0.392***	0.006	0.321***	0.057	-0.327***	0.209***	0.303***	-0.138***	—		
BI	Spearman's Rho	-0.960***	0.711***	0.448***	0.883***	0.287***	0.124**	-0.280***	-0.521***	0.479***	0.546***	0.118**	0.180***	0.187***	-0.154***	0.102*	0.633***	0.116**	0.165***	0.035	0.203***	0.762***	0.131**	0.227***	—	
pH	Spearman's Rho	0.085*	-0.233***	-0.142***	-0.323***	0.367***	0.158***	0.385***	0.482***	0.210***	-0.458***	0.110**	-0.121**	0.163***	0.024	0.334***	0.107**	-0.457***	-0.151***	0.415***	-0.301***	-0.186***	0.143***	-0.460***	-0.153***	—

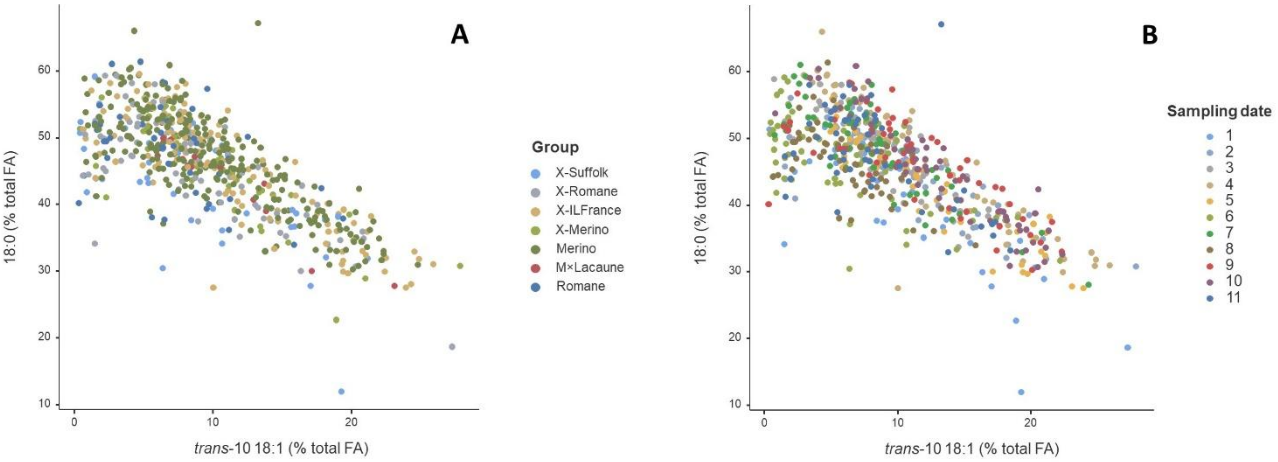

Figure S3 - Relationships between *trans*-10 18:1 and 18:0 (% of total fatty acids). Panel A shows data colored by genetic group, and Panel B shows data colored by Sampling date. Each point represents an individual animal.

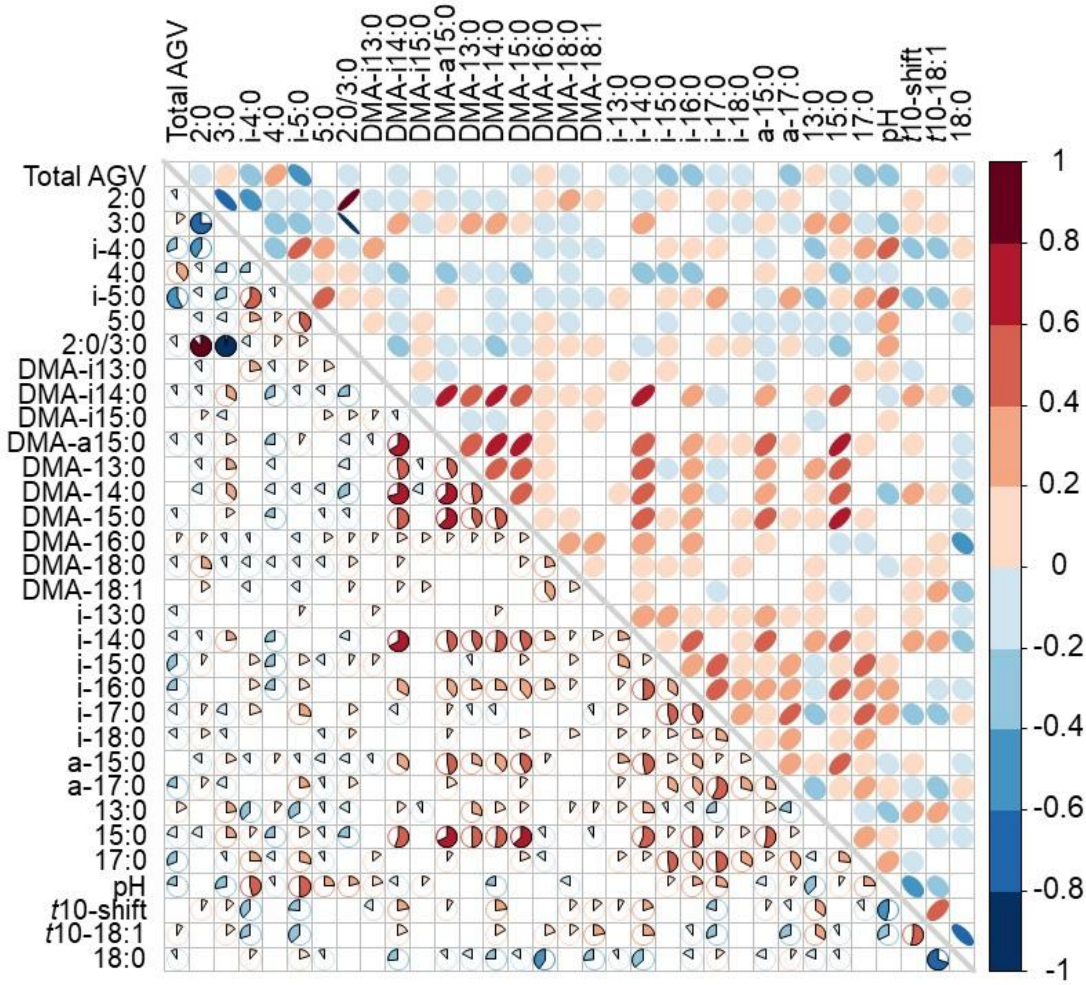

Figure S4 - Spearman's rank correlation matrix among volatile fatty acids (VFA), dimethyl acetals (DMA), odd and branched chain fatty acids, as well as pH, *t*10-shift, *t*10-18:1, and 18:0 in the rumen content. Color intensity and ellipse orientation indicate the strength and direction of correlations (red = positive; blue = negative). Only significant correlations (P < 0.05) are shown as filled ellipses.
